# Noncanonical projections to the hippocampal CA3 regulate spatial learning and memory by modulating the feedforward hippocampal trisynaptic pathway

**DOI:** 10.1371/journal.pbio.3001127

**Published:** 2021-12-20

**Authors:** Xiaoxiao Lin, Michelle Amalraj, Crisylle Blanton, Brenda Avila, Todd C. Holmes, Douglas A. Nitz, Xiangmin Xu

**Affiliations:** 1 Department Anatomy & Neurobiology, School of Medicine, University of California, Irvine, California, United States of America; 2 Department Physiology & Biophysics, School of Medicine, University of California, Irvine, California, United States of America; 3 The Center for Neural Circuit Mapping, University of California, Irvine, Irvine, California, United States of America; 4 Department of Cognitive Science, University of California San Diego, La Jolla, California, United States of America; AUSTRIA

## Abstract

The hippocampal formation (HF) is well documented as having a feedforward, unidirectional circuit organization termed the trisynaptic pathway. This circuit organization exists along the septotemporal axis of the HF, but the circuit connectivity across septal to temporal regions is less well described. The emergence of viral genetic mapping techniques enhances our ability to determine the detailed complexity of HF circuitry. In earlier work, we mapped a subiculum (SUB) back projection to CA1 prompted by the discovery of theta wave back propagation from the SUB to CA1 and CA3. We reason that this circuitry may represent multiple extended noncanonical pathways involving the subicular complex and hippocampal subregions CA1 and CA3. In the present study, multiple retrograde viral tracing approaches produced robust mapping results, which supports this prediction. We find significant noncanonical synaptic inputs to dorsal hippocampal CA3 from ventral CA1 (vCA1), perirhinal cortex (Prh), and the subicular complex. Thus, CA1 inputs to CA3 run opposite the trisynaptic pathway and in a temporal to septal direction. Our retrograde viral tracing results are confirmed by anterograde-directed viral mapping of projections from input mapped regions to hippocampal dorsal CA3 (dCA3). We find that genetic inactivation of the projection of vCA1 to dCA3 impairs object-related spatial learning and memory but does not modulate anxiety-related behaviors. Our data provide a circuit foundation to explore novel functional roles contributed by these noncanonical hippocampal circuit connections to hippocampal circuit dynamics and learning and memory behaviors.

## Abstract 2

This study reveals extensive non-canonical synaptic inputs to dorsal hippocampal CA3 from ventral CA1, perirhinal cortex and subicular complex, and shows that genetic inactivation of projection from ventral CA1 to dorsal CA3 impairs object-related spatial learning and memory.

## Introduction

The extended hippocampal formation (HF) includes the entorhinal cortex (EC), dentate gyrus (DG), cornu ammonis (CA1, CA2, and CA3), and the subiculum (SUB) complex. Much of the seminal work on hippocampal connectivity was carried out using conventional chemical tracing techniques [1–5]. The HF is traditionally characterized as having a feedforward, unidirectional circuit organization [6–8]. Based on the trisynaptic pathway model, the CA1 transfers excitatory information out of the hippocampus proper to SUB, which has been traditionally considered as a second major output stage of HF [8–11]. While this canonical HF connectivity has been well established, new viral genetic circuit mapping approaches have revealed noncanonical HF circuits.

Our recent studies [12–14] using genetically modified rabies virus–based retrograde tracing show a significant back projection pathway from SUB to hippocampal CA1 in the mouse. This SUB back projection pathway (SUB-CA1) is “noncanonical” as it runs directionally opposite the prominent feedforward pathway from CA1 to SUB. Functionally, the SUB back projection pathway contributes critically to object–place learning and to spatial tuning of neuronal activity in CA1 [12]. In contrast to the feedforward pathway from CA3 to CA1 to SUB, hippocampal theta frequency (8 Hz) network oscillations can flow “in reverse” from SUB to CA1 and CA3 to modulate spike timing and local network rhythms in these subregions [15–17]. The large extent of SUB activity back propagation suggests an even larger, noncanonical circuit network involving the subicular complex, hippocampal CA1 and CA3.

CA3 receives 3 major converging inputs: the mossy fiber input from DG granule cells, EC input via the perforant path, and the extensive recurrent collateral inputs from other CA3 neurons [3]. There is known structural heterogeneity of CA3 subregions along the transverse axis from proximal CA3 (CA3c) through middle CA3 (CA3b) to distal CA3 (CA3a). The distribution of CA3 anatomical inputs gradually changes along the transverse axis, as mossy fiber inputs from the DG taper off in the proximodistal direction [3,18,19]. By contrast, 2 connectivity gradients increase with associational projections between CA3 excitatory cells becoming more prominent and projections from EC layer II cells also becoming stronger along proximal to distal locations [9,20,21].

Gene expression markers and their distribution patterns have been used to distinguish up to 9 CA3 domains according to their proximodistal and septotemporal locations [22]. Domains #3, 2, and 1 correspond to the CA3a, b, and c in septal regions of CA3 as first proposed by Lorente de No. Domains #4 to 6 span approximately the midseptotemporal half (from proximal to distal), and domains #7 to 9 cover the temporal pole of CA3. Physiological and functional studies reveal CA3 subregional differences corresponding to their structural and molecular expression heterogeneity along the transverse and septotemporal axes [23–25].

While classical studies provide an overall understanding of circuit connectivity for CA3, noncanonical circuit connections have not been studied, and quantitative examinations of intrinsic and extrinsic inputs (including noncanonical connections) to the subregions of CA3 are lacking. In this study, we performed multiple sets of viral tracing experiments using retrograde genetically modified canine adenovirus type 2 (CAV2) [26–28], a new designer variant of adenovirus-associated virus (rAAV2-retro) [29,30] and rabies virus [12–14,31], as well as anterograde herpes simplex virus (HSV, H129 strain) [32,33]. We find substantial noncanonical synaptic inputs to dorsal hippocampal CA3 from ventral CA1 (vCA1), perirhinal cortex (Prh), and SUB complex including ventral subiculum (SUBv) and subiculum transition area (SUBtr). In particular, the input from vCA1 to dorsal CA3 (dCA3) runs opposite the trisynaptic pathway and opposite the direction of flow of the predominant theta frequency field potential oscillation along the septotemporal axis. These reverse, noncanonical pathways might provide a feedback regulation for CA3 that may augment the trisynaptic circuit connections and complement and strengthen CA3 autoassociative connections. We performed chemogenetic manipulations and tested the behavioral functions of vCA1 to dCA3 pathway using object location memory (OLM), novel object recognition, and anxiety-related tests. We find that inhibition of dCA3-projecting vCA1 neurons impairs location and object-related memory but not the anxiety behaviors. These findings lay a strong foundation to consider the largely unexplored functional roles of these noncanonical hippocampal circuit connections.

## Results

### Noncanonical inputs to dorsal CA3 revealed by CAV2-Cre and rAAV2-retro-Cre tracing

**Fig 1:**
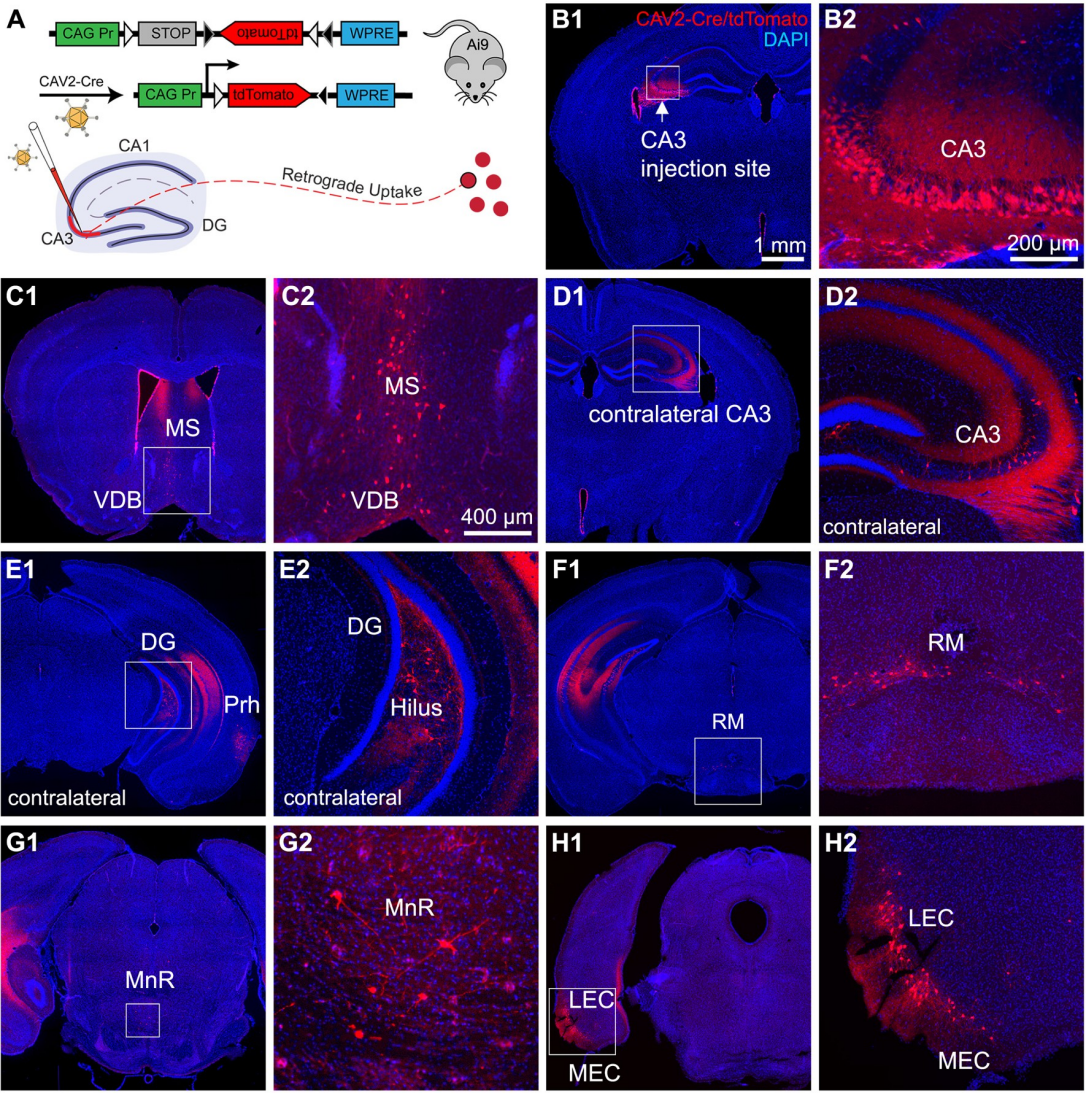
Retrograde labeling by canine adenovirus 2 (CAV2-ΔE1-Cre) reveals canonical circuit inputs to dorsal hippocampal CA3. **(A)** Schematic for retrograde monosynaptic circuit tracing using E1-deleted CAV2 viral vector expressing the Cre recombinase (CAV2-Cre) in Ai9 tdTomato Cre-reporter mice. Top: A loxP-flanked STOP cassette preventing transcription of a CAG promoter-driven RFP variant (tdTomato) inserted into the Gt (ROSA)26Sor locus of the Ai9 mouse genome. Following Cre-mediated recombination, cells in Ai9 mice express robust tdTomato fluorescence signals. Bottom: CAV2-Cre injection into dorsal hippocampal CA3 leads to the monosynaptic retrograde tracing of circuit inputs to the CA3 region in the Ai9 mouse and RFP labeling near the injection site. **(B)** Representative section images of viral injection sites in the hippocampal CA3. B1: The CA3 region is indicated by a white arrow. DAPI staining is blue. B2: High magnification view of the injection site. CAV2-infected cells are labeled by tdTomato in the pyramidal cell layer of CA3. **(C–H)** Results of CAV2-based retrograde monosynaptic circuit tracing from dCA3. For all panel sets, the labeled region is indicated by the white box (left), then an enlarged view is shown on the right. CA3 inputs include the MS and VDB (C1 and C2), contralateral CA3 (D1 and D2), contralateral dentate hilus (E1 and E2), RM nucleus (F1 and F2), MnR (G1 and G2), and LEC and MEC (H1 and H2). The scale bar (1 mm) applies to the lower magnification panels B1, C1, D1, E1, F1, G1, and H1; the scale bar (400 μm) applies to higher magnification panels C2, D2, E2, F2, and H2; and the scale bar (200 μm) applies to higher magnification panels B2 and G2. dCA3, dorsal CA3; DG, dentate gyrus; LEC, lateral entorhinal cortex; MEC, medial entorhinal cortex; MnR, median raphe nucleus; MS, medial septum; RFP, red fluorescent protein; RM, retromammillary; VDB, vertical diagonal band.

To map brain-wide circuit inputs to hippocampal CA3, we injected a monosynaptic retrograde viral tracer, E1-deleted canine adenovirus 2, which expresses Cre-recombinase (CAV2-ΔE1-Cre) [27] into dorsal hippocampal CA3 in Cre-dependent Ai9 reporter mice for visualization of retrogradely traced neuron with their tdTomato expression (Fig 1A). This recombinant CAV2 vector is replication incompetent due to the lack of a critical E1 gene from the viral genome. Furthermore, this vector codes for Cre recombinase expression. Based on previous studies and our own applications [12,27,28], this Cre expressing virus robustly infects neurons in vivo and activates transgenic tdTomato expression in local and long-range connected populations of neurons in Cre-dependent reporter mouse lines.

Following CAV2-Cre injection in the Ai9 mice (*n* = 6 mice), tdTomato expression is seen in the local neurons in the dCA3 injection site as well as the presynaptic neurons that provide inputs to the CA3 injection site. Note that local CAV2-Cre labeled neurons are spatially restricted to the pyramidal layer of dCA3 (extending from CA3b to CA3a) without labeling DG or CA2/CA1; thus, putative excitatory CA3 neurons are specifically labeled (Fig 1B). Our staining data show that only 1% of CAV2-Cre labeled neurons (with a sample of 435 neurons from 4 sections of 2 mice) in the dCA3 injection site are immunopositive for GABA (S1A Fig). This is consistent with our published finding showing that CAV2 is taken up by excitatory projection neurons in the HF [12]. While CAV2 infects epithelial cells lining the lateral ventricle (Fig 1B), this does not lead to unexpected results. Our results confirm that dCA3 receives canonical inputs from the brain regions as identified from previous studies [3], including labeled neurons in the medial septum and diagonal band of Broca (MS-DBB), contralateral CA3, dentate hilus, and the entorhinal cortex (medial entorhinal cortex [MEC] and lateral entorhinal cortex [LEC]) (Fig 1C–1E, and 1H). A great majority of MEC and LEC input labels are located in layer II. Labeled neurons are also found in the retromammillary (RM) nucleus and the median raphe nucleus (MnR) (Fig 1F and 1G).

**Fig 2:**
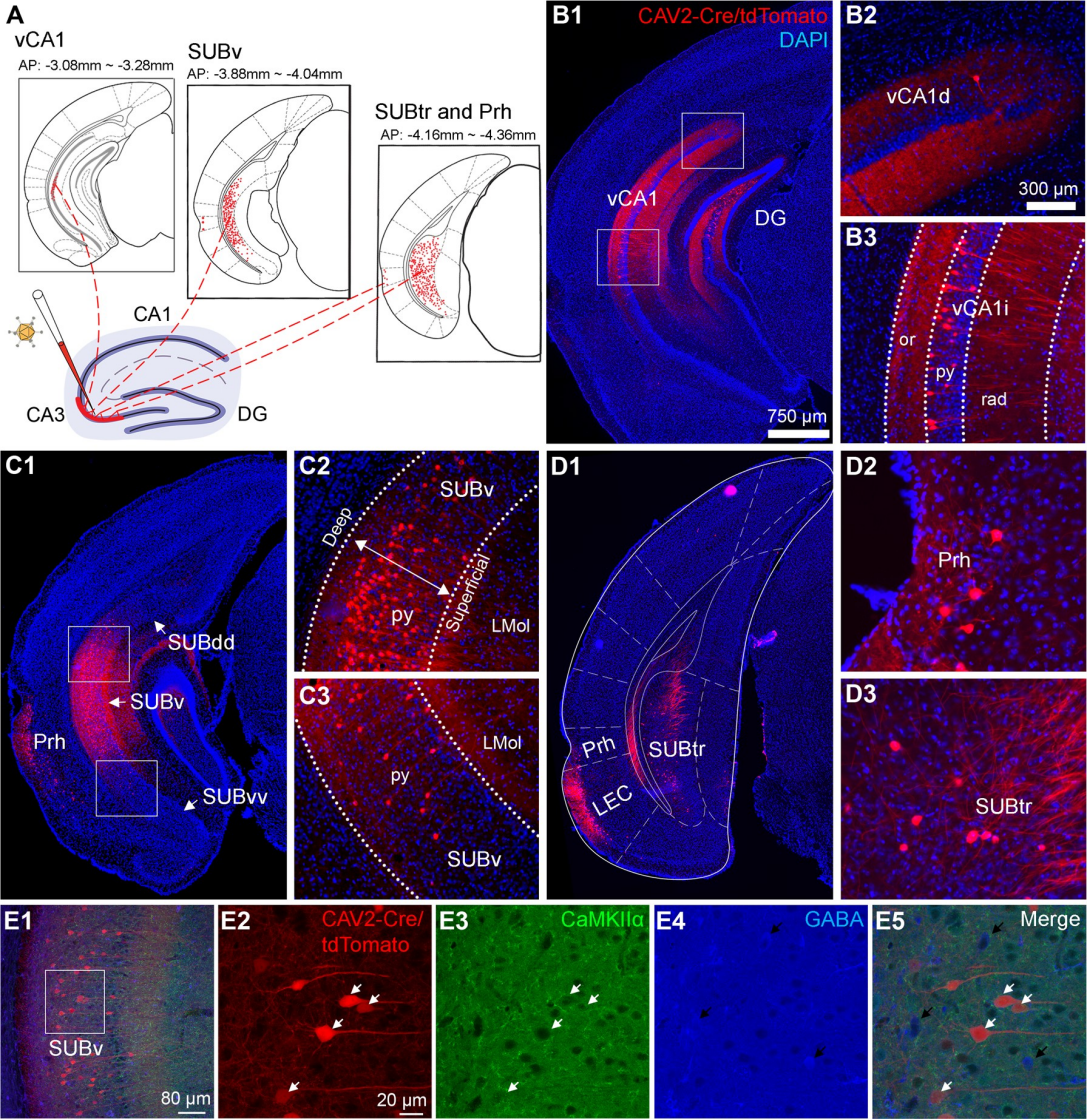
Retrograde labeling by canine adenovirus 2 (CAV2-ΔE1-Cre) reveals noncanonical circuit inputs from ventral hippocampal CA1, ventral subicular complex, and Prh to dorsal hippocampal CA3. **(A)** Results of CAV2-based retrograde monosynaptic circuit tracing from dCA3 and noncanonical input mapped regions including (from left to right): vCA1, the designated SUBv, and the SUBtr and Prh. Retrogradely labeled neurons are depicted in red. The AP coordinates from the Bregma are provided. **(B)** Detailed view of retrogradely labeled cells in vCA1. B1: The vCA1 input regions are indicated by white boxes. Enlarged views are shown in B2 and B3. **(C)** and **(D)** are similarly formatted as B, illustrating retrogradely labeled cells in SUBv, SUBtr, and Prh. **(E)** CAV2-labeled neurons in the presynaptic input regions of dCA3. E1 to E5: Example images of CaMKIIα and GABA immunostaining of CAV2-labeled presynaptic neurons in SUBv. A small region of interest in the overlay image (E1) is enlarged to show specific information in E2-5. CAV2-labeled neurons are visualized by tdTomato expression in E2. CaMKIIα immunoreactivity is visualized with a Cy5-conjugated secondary antibody, depicted in E3 as a green pseudo-color. GABAergic immunoreactivity is visualized with an AF 488-conjugated secondary antibody, depicted in E4 as a blue pseudo-color. White arrows indicate the colocalization of CAV2-labeled neurons and CaMKIIα+ immunostaining in E5. Black arrows point to GABA-positive neurons in E4. The scale bar (750 μm) applies to B1, C1, and D1; the scale bar (300 μm) applies to B2, B3, C2, C3, D2, and D3; the scale bar (800 μm) applies to E1; and the scale bar (20 μm) applies to E2 to E5. AP, anterior–posterior; CAV2, canine adenovirus type 2; dCA3, dorsal CA3; DG, dentate gyrus; LMol, lacunosum moleculare layer of the subiculum; Prh, perirhinal cortex; py, pyramidal cell layer; rad, radium layer of the hippocampus; SUBdd, dorsal subiculum; SUBtr, subiculum transition area; SUBv, ventral subiculum; SUBvv, the end tip of ventral subiculum; vCA1d: ventral CA1, dorsal (top) portion; vCA1i, ventral CA1, intermediate portion.

**Fig 3:**
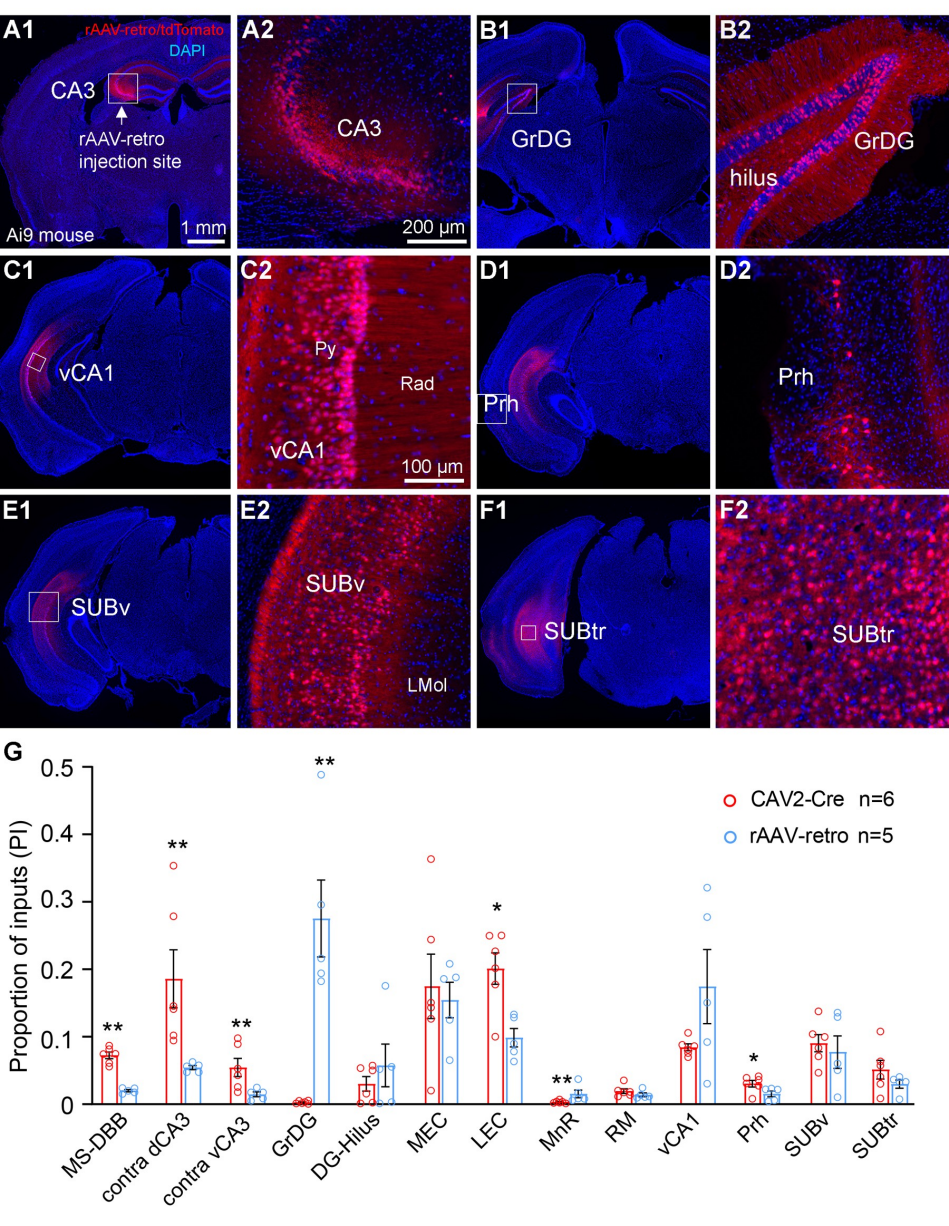
Retrograde labeling by rAAV2-retro-Cre shows canonical and noncanonical inputs to dorsal hippocampal CA3 in the Ai9 mouse. **(A)** Representative section images of the rAAV2-retro-Cre injection site in dorsal hippocampal CA3. A1: The CA3 injection site is indicated by a white arrow, within the white box. DAPI staining is blue. A2: High magnification view of the injection site. rAAV-retro infected cells are labeled by tdTomato in the CA3 region. **(B)** Labeling in the granular cell layer of the dentate gyrus (GrDG) (B1) and the high magnification view of the cell labels (B2). **(C–F)** CA3 noncanonical input sources identified with rAAV2-retro-Cre, lower magnification views in panels on the left and higher magnification views in panels on the right. The enlarged region of interest is from the white boxed area in the left panel. Noncanonical input regions include the vCA1 (C1 and C2), the Prh (D1 and D2), the SUBv (E1 and E2), and the SUBtr (F1 and F2). **(G)** Quantitative distribution of CAV2-Cre (*n* = 6 mice) and rAAV2-retro-Cre (*n* = 5 mice) labeled neurons. The PI index is calculated by the number of labeled neurons in each brain structure compared to the total number of labeled neurons from all input mapped regions except the local ipsilateral CA3 labels. The total number of input mapped neurons are on average, 4,862 ± 1,292.8 cells for CAV2-Cre cases (*n* = 6 mice) and 14,141 ± 4,618.2 cells for rAAV-retro cases (*n* = 5 mice). Each circle represents a PI data point from one mouse. All data are presented as mean ± SE; *, ** indicate the PI statistical significance level of *p* ≤ 0.05 and *p* ≤ 0.01 (Mann–Whitney U test), respectively. The raw data for Fig 3G are included in S1 Data. The scale bar (1 mm) in A1 applies to the low magnification images of A–F; the scale bar in A2 (200 μm) applies to A2, B2, D2, E2, and F2; and the scale bar (100 μm) applies to C2. LEC, lateral entorhinal cortex; MEC, medial entorhinal cortex; MnR, median raphe nucleus; MS-DBB, medial septum and diagonal band of Broca; PI, proportion of inputs; Prh, perirhinal cortex; RM, retromammillary; SUBtr, subiculum transition area; SUBv, ventral subiculum; vCA1, ventral CA1.

Using this retrograde viral tracing approach, we additionally identify substantial noncanonical inputs to dCA3, including retrogradely labeled neurons observed in vCA1 [anterior–posterior (AP) distance from Bregma: −3.08 mm to −3.28 mm] (Fig 2A). There are no input mapped neurons in dorsal CA1 (AP: −0.94 mm to −2.18 mm). We followed a published mapping study using gene expression markers to divide vCA1 into the dorsal (vCA1d), intermediate (vCA1i), and ventral (vCA1v) subdivisions along the septotemporal axis [22]. As depicted in S5A Fig, the ventral edge of the DG lateral blade is the same level as the border between vCA1d and vCA1i; the border of vCA1i and vCA1v is parallel to the dorsal edge of the rhinal fissure. We calculate the proportion of inputs (PI) index as the number of labeled presynaptic neurons in a brain region of interest versus the overall total labeled neurons in each case (CAV2-Cre, average total labeled neurons: 4,862 ± 1,292.8 (mean ± SE) cells per mouse, *n* = 6 mice). A number of vCA1 labeled neurons are located at the pyramidal layer of the lower portion of vCA1d, and intermediate vCA1 (vCA1i) (on average 430 ± 137.2 cells measured from 4 to 5 sections per mouse, *n* = 6 mice, PI = 0.084 ± 0.005) (Figs 2B and 3G). Only a few neurons are found in the upper portion of vCA1 (vCA1d) (on average 24 ± 7.0 cells measured from 4 to 5 sections per mouse, *n* = 6 mice, PI = 0.001) (Fig 2B2).

The CAV2-Cre tracing also reveals strong circuit inputs to dCA3 from the designated SUBv (S5B Fig) and the SUBtr (S5C Fig) based on Franklin and Paxinos’ mouse brain atlas [34] and recent anatomical mapping studies [35–37]. To accurately map the anatomical locations of CAV2-Cre labeled neurons, we use the SUB subregional terminology according to an earlier SUB gene expression mapping study [35] and distinguish the subicular complex subregions into the “classical” dorsal subiculum as SUBdd (aka dSUB or SUB), the intermediate portion of the ventral subiculum as SUBv, and the end tip of the ventral subiculum as SUBvv (Fig 2C and S5B Fig). SUBtr is close to the presubiculum (PrS), parasubiculum (PaS), and MEC (Fig 2D and S5C Fig).

The majority of CAV2-labeled neurons are located at the top and intermediate portions of SUBv (close to the SUBdd region) (on average 484 ± 188.9 cells measured from 3 to 4 sections per mouse, *n* = 6 mice, PI = 0.09 ± 0.013) (Figs 2C and 3G). A few labeled neurons are also observed at the bottom of SUBv (close to SUBvv) (on average 9 ± 5.1 cells measured from 3 to 4 sections per mouse, *n* = 6 mice, PI < 0.0001). In comparison with the superficial pyramidal layer of SUBv, more labeled neurons are spatially localized at the deeper pyramidal layer of SUBv (Fig 2C). SUBtr inputs are spatially located at the top and middle parts of the structure (on average 240 ± 112.3 cells measured from 3 to 4 sections per mouse, *n* = 6 mice, PI = 0.050 ± 0.014) (Figs 2D and 3G). The putative excitatory inputs are confirmed by our finding that all of CAV2-labeled vCA1, SUBv, and SUBtr neurons are negative for GABA immunostaining, and 98% of them are immunopositive for the excitatory cell marker calmodulin-dependent protein kinase IIα (CaMKIIα) (*n* = 904 cells for vCA1, 414 cells for SUBv and 764 cells for SUBtr, pooled from 4 sections of 2 mice for each region) (Fig 2E, S1B and S1C Fig). In addition to vCA1, SUBv and SUBtr inputs, we identify a direct projection from Prh to dCA3; none of the 45 labeled cells are GABA positive in Prh (from 4 sections of 2 mice) (Fig 2D, S1D Fig). Thus, CAV2-Cre–mediated retrograde mapping reveals multiple noncanonical input pathways from vCA1, subregions of subicular complex (SUBv and SUBtr), and Prh to dCA3 that have not been reported previously.

The finding of noncanonical inputs to dCA3 by CAV2-Cre was replicated using a designer AAV variant (rAAV2-retro-Cre) that permits efficient retrograde tracing of projection neurons (Fig 3; average total number of labeled neurons: 14,141 ± 4,618.2 cells per mouse, *n* = 5 mice). Following injection into dCA3 of the Ai9 mice, rAAV2-retro-Cre labels about 3 times the number of neurons labeled by CAV2-Cre. Both dentate granule cells and hilar cells are labeled in the rAAV2-retro-Cre experiments (Fig 3), while CAV2-Cre sparsely labels dentate granule cells (the number of labeled neurons in the dentate granule cell layer (GrDG), CAV2-Cre, 18 ± 8.6 cells per mouse, *n* = 6 mice; rAAV-retro-Cre, 3,330 ± 1,148.3 cells per mouse, PI = 0.275 ± 0.057, *n* = 5 mice; *p* = 0.004, Mann–Whitney U test) (Fig 3B and 3G). In addition to the observed DG labeling, we compared the retrograde labeling in other presynaptic input regions. Proportionally, we find CAV2-Cre labels more of the presynaptic neurons in MS-DBB, contralateral CA3, Prh, and LEC, whereas rAAV-retro-Cre shows higher proportions of labeling in MnR regions (Fig 3G). These variances in results indicate viral tropism effects [38–40].

### Monosynaptic rabies virus tracing of noncanonical circuit inputs to dorsal CA3

**Fig 4:**
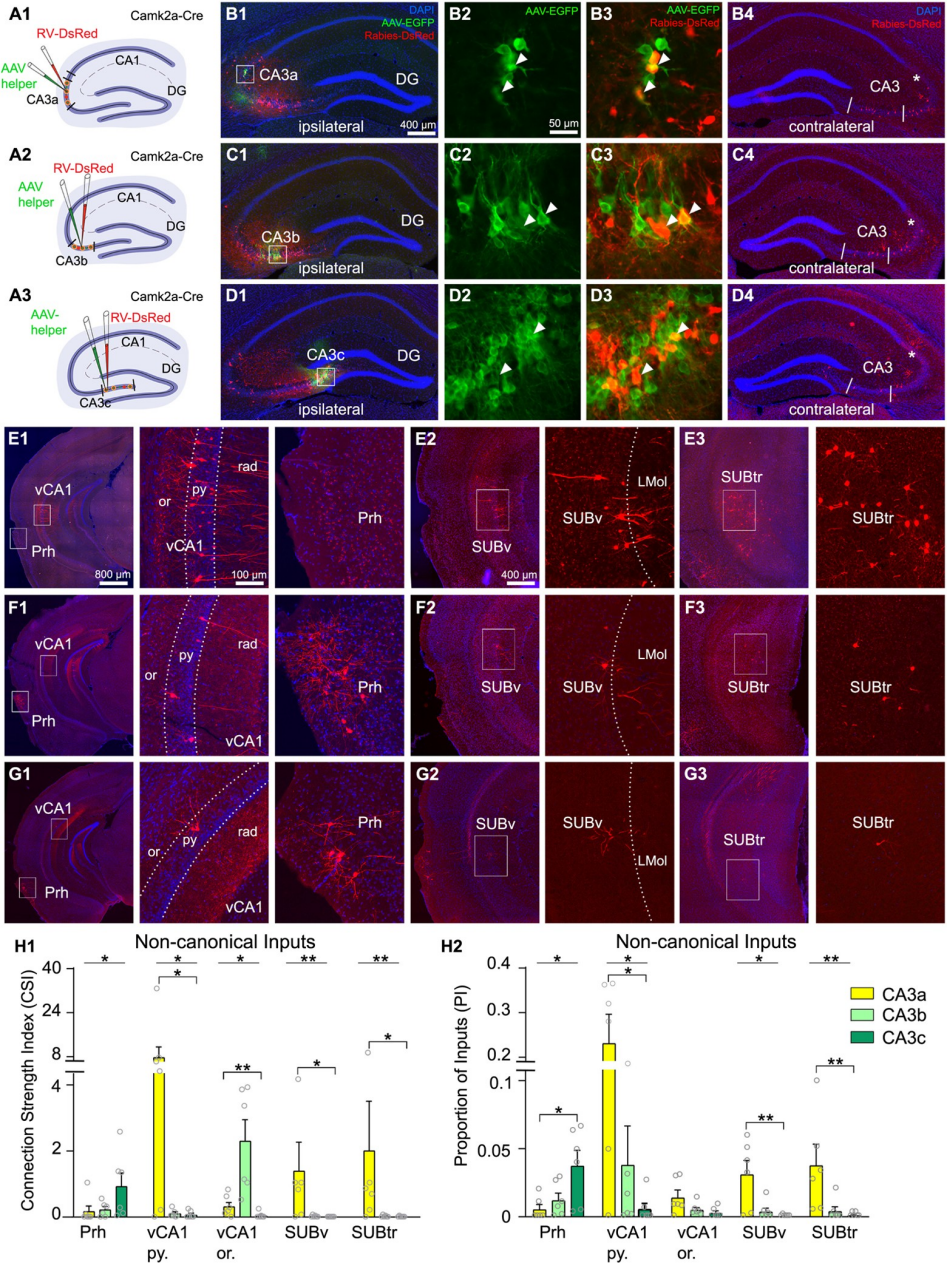
Cre-dependent retrograde monosynaptic rabies tracing reveals semiquantitative noncanonical CA3 inputs from vCA1, SUBv, Prh, and SUBtr. **(A)** Schematic of retrograde monosynaptic rabies tracing from dCA3 subregions in Cre transgenic mice. Injections of rabies virus (RV-DsRed: EnvA-SADΔG-RV-DsRed, red) and AAV helper virus (AAV8-hSyn-DIO-TC66T-2A-eGFP-2A-OG, green) are administered into specific subregions of dCA3. From top to bottom, the schematics of CA3a, CA3b, and CA3c injection sites are shown. **(B–D)** Images of injection sites of the 3 CA3 subregions (B1 to B3 for CA3a, C1-C3 for CA3b and D1 to D3 for CA3c) and corresponding rabies virus–mediated labeling of presynaptic neurons in contralateral CA3 regions with DAPI staining (B4, C4, and D4). Injection sites are shown in white boxes along the transverse axis. The enlarged views of the white box regions are shown for CA3a (B2 and B3), CA3b (C2 and C3), and CA3c (D2 and D3). Images in B2, C2, and D2 show EGFP-labeled excitatory cells (green), while the images of B3, C3, and D3 allow for visualization of DsRed and EGFP double-labeled starter neurons (white arrowheads). The images of B4, C4, and D4 show contralateral CA3 inputs for the corresponding dCA3 subregions. The star indicates the border of CA3a and CA2 determined by PCP4 staining (S3 Fig). The boundaries of 3 CA3 subregions are indicated by white lines. The scale bar (400 μm) applies to B1, B4, C1, C4, D1, and D4. The scale bar (50 μm) applies to B2, B3, C2, C3, D2, and D3. **(E)** Illustration of noncanonical inputs to the CA3a subregion (E1 to E3). The boxed regions at vCA1 and Prh in the E1 left panel are shown in the middle and right panels, respectively, at a higher magnification. The cell labels in the SUBv and SUBtr are shown in E2 and E3. **(F)** and **(G)** are formatted similarly to **E**, to illustrate noncanonical inputs to CA3b and CA3c subregions, respectively, from vCA1, Prh, SUBv, and SUBtr. The scale bar (800 μm) applies to E1, F1, and G1. The scale bar (400 μm) applies to E2, F2, G2, E3, F3, and G3. The scale bar (100 μm) applies to all the magnified input regions. **(H1)** Semiquantitative analyses of input connection strengths measured by the CSI across Prh, vCA1, SUBv, and SUBtr following rabies tracing in CA3 subregions. vCA1 inputs are organized by the spatial location at the pyramidal layer (vCA1 py.) and oriens layer (vCA1 or.). *n* = 6 mice per CA3 subregion. Data are from 8 Camk2α-Cre; TVA mice and 10 Camk2α-Cre mice. All data are presented as mean ± SE; *, ** indicate the CSI statistical significance level of *p* ≤ 0.05 and *p* ≤ 0.01, respectively (Kruskal–Wallis test followed by Dunn comparison test). The results of the Kruskal–Wallis tests are indicated by the lines, and the results of Dunn tests are indicated by the brackets above the bars. See S2 and S3 Tables for more statistical information. **(H2)** Semiquantitative measurements of the PI for specific brain regions following the same format as in H1. The same abbreviations as in Fig 1. The raw data for Fig 4H1 and 4H2 are included in S2 Data. CSI, connection strength index; dCA3, dorsal CA3; DG, dentate gyrus; Prh, perirhinal cortex; SUBtr, subiculum transition area; SUBv, ventral subiculum; vCA1, ventral CA1.

Building on CAV2-Cre and rAAV2-retro-Cre tracing experiments, we conducted monosynaptic retrograde rabies virus–mediated tracing from dCA3. The monosynaptic rabies virus approach enables targeting of specific dCA3 subregions along the transverse axis. As rabies virus labeling has the feature of measurable starter cells at the injection site, connectivity strengths of input mapped brain regions per starter cell can be measured quantitatively. The rabies tracing system targets specific cell types using EnvA pseudotyping and limits transsynaptic spread to direct presynaptic inputs using glycoprotein gene-deleted (ΔG) rabies virus and trans-complementation [13,31,41]. Specifically, the ΔG rabies virus (deletion mutant, SAD-B19 strain) is pseudotyped with the avian sarcoma leucosis virus glycoprotein EnvA (EnvA-SADΔG rabies virus), which can only infect neurons that express the avian tumor virus receptor A (TVA). The TVA is an avian receptor protein that is absent in mammalian cells unless it is expressed through exogenous gene delivery. The deletion-mutant rabies virus can then be trans-complemented with the expression of rabies glycoprotein (RG) in the same TVA-expressing cells to enable its retrograde spread restricted to direct presynaptic neurons. Because these presynaptic neurons lack RG expression, the virus cannot spread further beyond these cells. We used Camk2a-Cre (T29) mice [42] to target excitatory CA3 neurons for Cre-dependent rabies tracing. In Camk2a-Cre or double-transgenic mice (Camk2a-Cre; TVA), we virally traced circuit connections to a small population of CA3 starter cells located in different CA3 subregions (average number of starters per section: CA3a, 5 ± 1.1 neurons, *n* = 6 mice; CA3b, 7 ± 1.7 neurons, *n* = 6 mice; CA3c, 8 ± 2.4 neurons, *n* = 6 mice). The starter cells are unambiguously identified by their EGFP and DsRed expression from the helper AAV and ΔG-DsRed rabies genomes, respectively (Fig 4A–4D, S2 Fig). Note that qualitatively similar noncanonical inputs to CA3 were shown for 2 slightly different approaches using Camk2a-Cre; TVA mice (*n* = 8) injected with the helper AAV8-EF1a-DIO-H2B-GFP-2A-oG or using the Camk2a-Cre mice (*n* = 8) injected with the helper AAV8-hSyn-DIO-TC66T-2A-eGFP-2A-oG. The efficiency of different helper AAV infection measured by GFP labeled cells in injection sites tends to be similar (S2 Fig). We pooled data from these experiments for quantitative analyses of input connectivity strengths.

**Fig 5:**
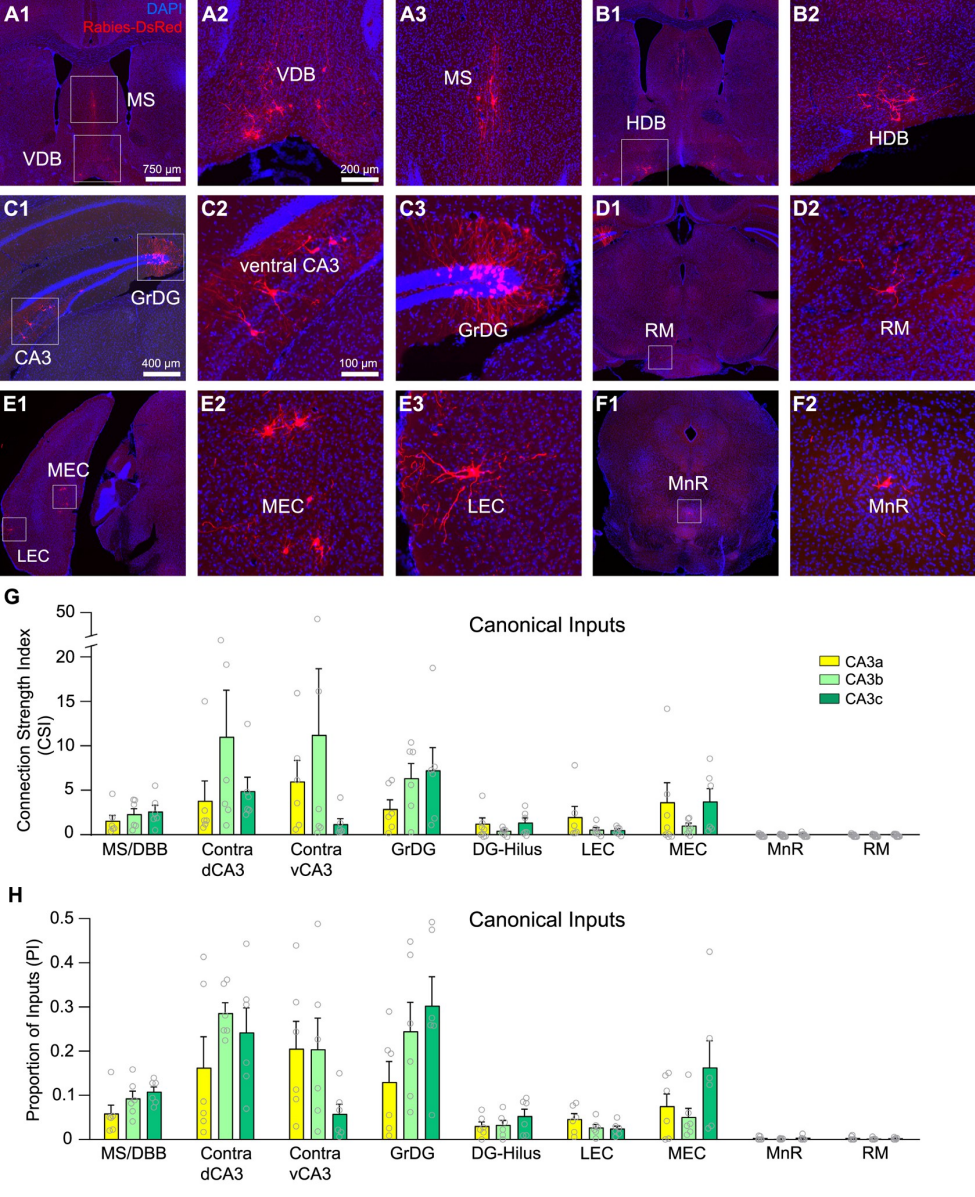
Cre-dependent monosynaptic rabies tracing reveals local and distant circuit input connections to CA3a, CA3b, and CA3c subregions. **(A–F)** Example images showing monosynaptic rabies virus–labeled neurons in presynaptic input regions. Presynaptic cells are found in the MS, the VDB of Broca (A1 to A3), the HDB of Broca (B1 and B2), ventral CA3, the GrDG (C1 to C3), RM nucleus (D1 and D2), LEC, MEC (E1 to E3), and the MnR (F1 and F2). **(G)** Quantitative measurements of input connection strengths by the CSI following rabies tracing from CA3a, CA3b, or CA3c. The data were measured from 8 CaMK2α-Cre; TVA mice and 10 Camk2α-Cre mice with *n* = 6 mice per CA3 subregion. All data are presented as mean ± SE. **(H)** Measurements of the PI for specific brain regions following rabies tracing from CA3a, CA3b, or CA3c. See S2 and S3 Tables for further details. The raw data for Fig 5G and 5H are included in S2 Data. The scale bar (750 μm) applies to A1, B1, D1, E1, and F1. The scale bar (400 μm) applies to C1. The scale bar (200 μm) applies to A2, A3, and B2. The scale bar (100 μm) applies to C2, C3, D2, E2, E3, and F2. GrDG, granule cell layer of the dentate gyrus; HDB, horizontal diagonal band; LEC, lateral entorhinal cortex; MEC, medial entorhinal cortex; MnR, median raphe nucleus; MS, medial septum; PI, proportion of inputs; RM, retromammillary; VDB, vertical diagonal band.

As shown in Fig 4A–4D, our rabies tracing location in more distal dCA3 is termed CA3a (corresponding to domain # 3 defined by Thompson and colleagues [22]). The more intermediate location of CA3 is termed CA3b (corresponding to domain # 2) and the more proximal dCA3 is termed CA3c (corresponding to domain # 1) [22]. As per our earlier publication [43], we stained hippocampal sections with Purkinje cell protein 4 (PCP4) antibody to delineate hippocampal CA2 (S3 Fig). Strong PCP4 immunoreactivity is localized in CA2; PCP4 immunostaining also results in concurrent detection of the mossy fiber tract, which allows for distinguishing distal CA3 from the CA2 region. The border of CA3/CA2 determined with PCP4 immunostaining matches the cytoarchitectural feature of the pyramidal layer identified by the DAPI staining (S3 Fig) [43]. The midline of the fimbria separates CA3a and CA3b [44]. CA3c is the subregion enclosed within DG. Similar to CAV2-Cre results, the afferent circuit inputs to dCA3 subregions originate from multiple brain regions that provide canonical input, including MS-DBB, DG, ventral CA3, EC, RM nucleus, and the MnR (Fig 5A–5F). Their measurements of quantitative input strengths for different dCA3 subregions are shown in Fig 5G and 5H and S2 and S3 Tables. We operationally define the input connection strength index (CSI) as the ratio of the number of presynaptic neurons in a brain region versus the number of starter neurons in the CA3 subregion. The CSI values allow us to quantitatively compare how input strengths from different brain regions to CA3 vary along the transverse axis. In addition, we calculate the PI index for comparison with CAV2-Cre and rAAV-retro-Cre retrograde viral tracers (Fig 5, S2 and S3 Tables).

To assess whether the noncanonical inputs have topographic gradients along the dCA3 transverse axis, we focus on comparing CSI and PI measurements of vCA1, SUBv, SUBtr, and Prh to different dCA3 subregions. We find that the more distally positioned neurons of dCA3 excitatory cells receive stronger excitatory inputs from the pyramidal layer of vCA1 (vCA1 py.) compared to the more proximally positioned neurons (CSI for vCA1 py.: CA3a = 9.35 ± 5.11, *n* = 6 mice; CA3b = 0.26 ± 0.11, *n* = 6 mice; CA3c = 0.07 ± 0.05, *n* = 6 mice. Kruskal–Wallis test, *p* = 0.036; Dunn comparison test, CA3a versus CA3c *p* = 0.037) (Fig 4E–4H, S2 and S3 Tables). The inhibitory inputs from the oriens layer of vCA1 (vCA1 or.) are also observed and their CSI values follow a similar trend to the pyramidal layer of vCA1 (CSI for vCA1 or.: CA3a = 0.33 ± 0.13; CA3b = 0.12 ± 0.05; CA3c = 0.05 ± 0.04. Kruskal–Wallis test, *p* = 0.046; Dunn comparison test, CA3a versus CA3c *p* = 0.005) (Fig 4H1, S2 and S3 Tables). The PI measurements follow the same trend as the CSI values for the CA3 subregions (PI for vCA1py.: CA3a = 0.237 ± 0.07, *n* = 6 mice; CA3b = 0.04 ± 0.03, *n* = 6 mice; CA3c = 0.005 ± 0.004, *n* = 6 mice; PI for vCA1 or.: CA3a = 0.014 ± 0.005; CA3b = 0.005 ± 0.002; CA3c = 0.002 ± 0.002) (Fig 4H2, S2 and S3 Tables). For direct comparison with canonical inputs, their quantitative strengths for different dCA3 subregions are shown in Fig 5G and 5H, but they do not show subregional differences.

Verifying the CAV2-Cre and rAAV2-retro-Cre tracing results, our rabies virus tracing data show that CA3 excitatory neurons receive inputs from SUBv and SUBtr regions. The superficial pyramidal neurons of the SUBv region have stronger connections with the excitatory neurons located at distal CA3 region, CA3a, compared to the ones at proximal region, CA3c (CSI for SUBv: CA3a = 1.41 ± 0.86, *n* = 6 mice; CA3b = 0.03 ± 0.01, *n* = 6 mice; CA3c = 0.002 ± 0.002, *n* = 6 mice. Kruskal–Wallis test, *p* = 0.009; Dunn comparison test, CA3a versus CA3c *p* = 0.014) (Fig 4E–4H, S2 and S3 Tables). Similarly, CA3a receive more presynaptic SUBtr inputs when compared with those received by CA3c (CSI for SUBtr: CA3a = 2.02 ± 1.48, *n* = 6 mice; CA3b = 0.03 ± 0.01, *n* = 6 mice; CA3c = 0.01 ± 0.01, *n* = 6 mice. Kruskal–Wallis test, *p* = 0.005; Dunn comparison test, CA3a versus CA3c *p* = 0.014) (Fig 4E–4H, S2 and S3 Tables). In addition, we find that the Prh inputs to CA3 subregions follow an opposite gradient arrangement, in which proximal CA3, CA3c, receives stronger Prh inputs compared to distal CA3, CA3a (CSI for Prh: CA3a = 0.181 ± 0.164, *n* = 6 mice; CA3b = 0.231 ± 0.090, *n* = 6 mice; CA3c = 0.942 ± 0.394, *n* = 6 mice. Kruskal–Wallis test, *p* = 0.049) (Fig 4E–4H, S2 and S3 Tables). Significant differences are observed in the PI measurements of SUBtr, SUBv, and Prh between CA3a and CA3c (Fig 4H2, S2 and S3 Tables).

### Anterograde H129-mediated tracing confirms vCA1-dCA3 and SUBv-dCA3 projections

**Fig 6:**
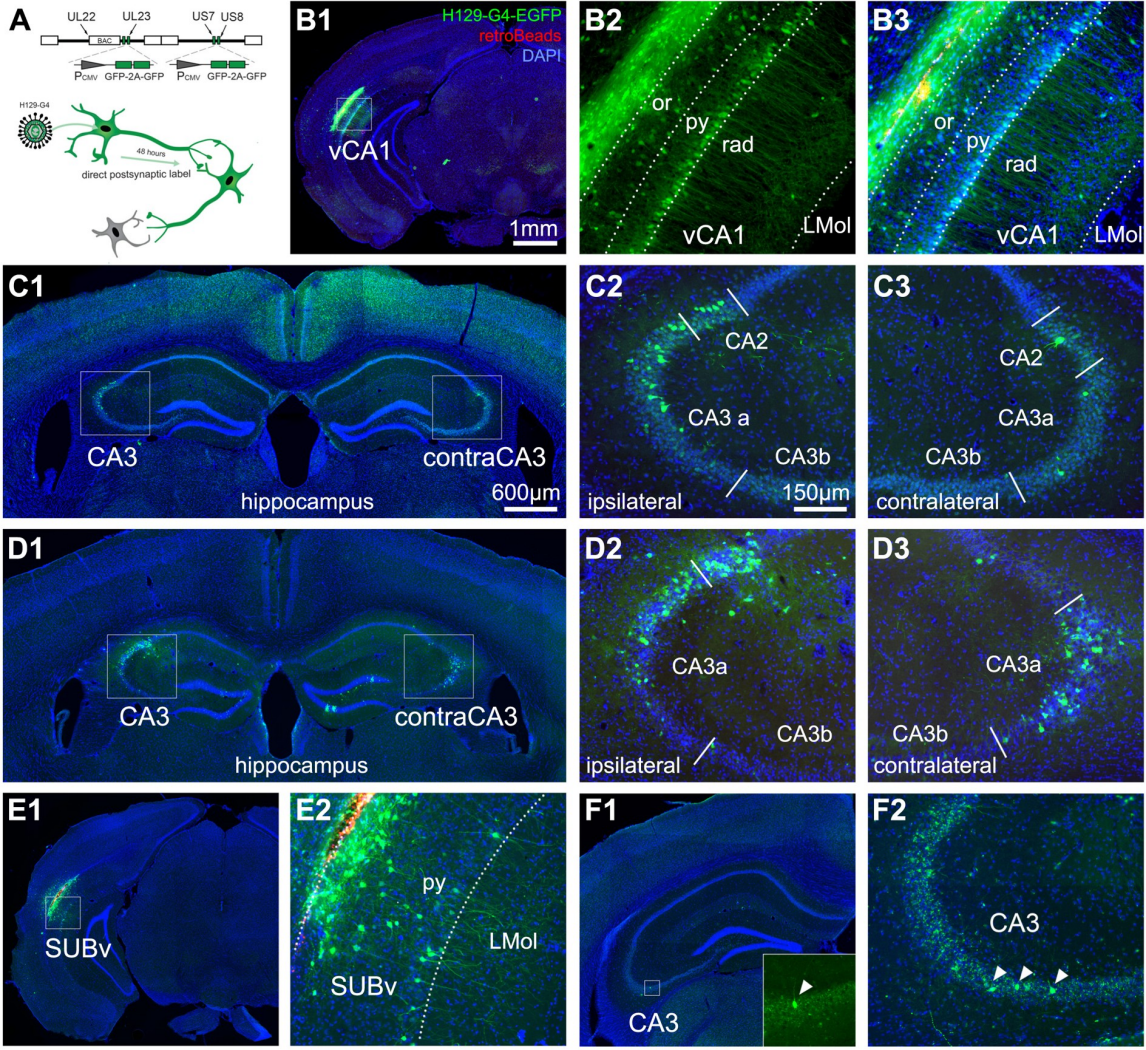
Anterograde HSV (H129 strain) tracing verifies direct projections from vCA1 and SUB to dCA3. **(A)** Schematic for anterograde H129-G4 for time-limited mapping of direct monosynaptic projections. Top: Illustration of the genetically modified H129-based viral vector with the insertion of 4 copies of EGFP (2 tandem EGFP cassettes) into the H129 genome. Bottom: Empirically determined timeline of propagation of H129-G4 for anterograde monosynaptic tracing. **(B)** Representative section images of the vCA1 injection site with H129-G4. B1: The H129-G4 injection site labeling (green = GFP, red = Retrobeads microspheres, blue = DAPI throughout all panels). B2: The EGFP-labeled neurons in the pyramidal layer (py) and oriens layer (or) of vCA1. B3: The merged image of H129-G4 expression and red microspheres that were co-injected with the H129 virus. **(C)** Representative section images for H129-G4 tracing from vCA1. C1: H129-G4 labeled neurons in dCA3 of both hemispheres. Enlarged views of the boxed regions in C1 are shown in C2 and C3. **(D)** Examples from a different case show H129-G4 tracing from vCA1. **(E, F)** Representative sections for H129-G4 tracing from SUBv. E1. The H129-G4 virus injection site. E2 shows the EGFP-labeled neurons in the py of SUBv. F1. H129-G4 labeled neurons in dCA3. An enlarged view of the boxed region in F1 is shown in the bottom right corner. F2. Verification of the noncanonical input by a different case of H129-G4 tracing from SUBv. The scale bar (1 mm) applies to B1 and E1; the scale bar (600 μm) applies to C1, D1, and F1; and the scale bar (150 μm) applies to B2, B3, C2, C3, D2, D3, E2, and F2. dCA3, dorsal CA3; HSV, herpes simplex virus.

In order to confirm the direct projections of vCA1 and SUBv to dCA3, we injected an anterograde-directed herpes virus (H129 strain; H129-G4) [33] to vCA1 or SUBv in different cohorts of wild-type C57BL/J6 mice. H129-G4 is generated by inserting binary, tandemly connected EGFP cassettes into the H129 genome. The EGFP fluorescent label of H129-G4 is sufficiently strong to visualize morphological details of labeled neurons (Fig 6A). We first injected H129-G4 into the pyramidal layer of vCA1 in the C57BL/J6 mice and used our empirically determined 48-hour incubation time to limit the anterograde monosynaptic transmission (Fig 6A and 6B; *n* = 4 mice). Our results show that H129-G4 labels ipsilateral and contralateral dCA3 neurons following the anterograde viral tracing from vCA1 (Fig 6C and 6D). The postsynaptic neurons are located at more distal CA3 (Fig 6C2 and 6D2). Our H129-G4 results also show a back projection from vCA1 to CA2 (Fig 6C and 6D), but the detailed neuronal connections and functions will need to be further examined in a future study.

To confirm the back projection from SUBv to CA3, a small amount of the H129-G4 virus was delivered and restricted to the SUBv region (*n* = 2 mice). We observe H129-G4–labeled SUBv neurons locally at the SUBv injection site (Fig 6E); the number of total labeled neurons in the SUBv injection site is less than the number of labeled neurons in vCA1 (*n* = 4 mice) (Fig 6B–6E). More sparsely labeled dCA3 neurons are found following the H129-G4 injection in SUBv (*n* = 2 mice) (Fig 6F).

Through anterograde and retrograde viral tracing experiments, our data demonstrate the existence of significant noncanonical circuit inputs from vCA1, SUB complex, and Prh to dCA3, which offers the anatomical circuit basis to explore functional roles of these noncanonical hippocampal circuit connections.

### Genetic inactivation of the ventral CA1 to dorsal CA3 projection impairs object-related spatial learning and memory

**Fig 7:**
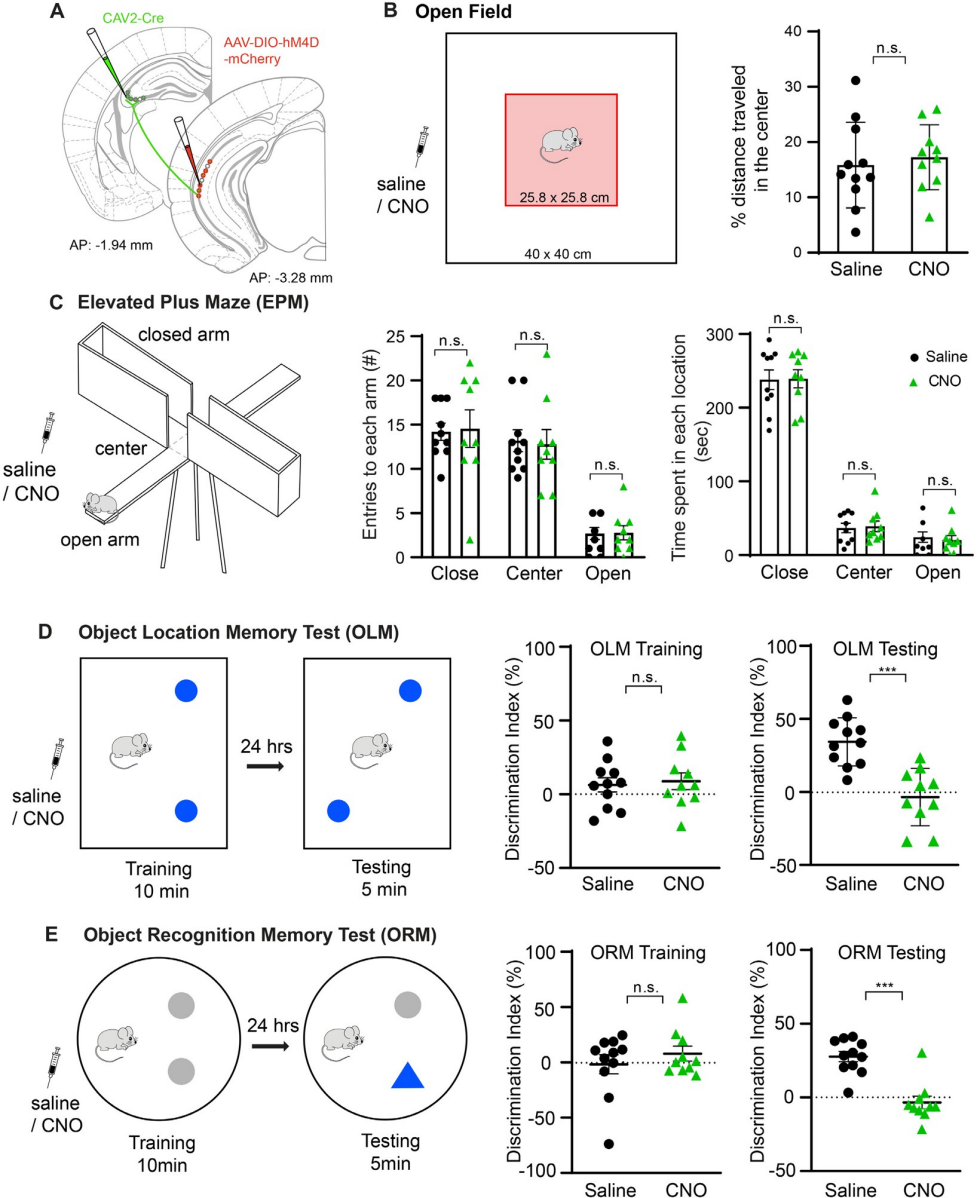
Genetic inactivation of the projection of vCA1 to dCA3 impairs object-related spatial learning and memory but does not modulate anxiety-related behaviors. **(A)** Schematic illustration of our strategy for genetic inactivation of dCA3-projecting vCA1 neurons with a bilateral injection of retrograde transporting CAV2-Cre in dCA3 and AAV2-DIO-hM4D-mCherry in vCA1. **(B)** Left, Illustration of the open field test. The box represents a locomotion test chamber (40 cm × 40 cm), and the pink shaded area depicts the center zone (25.8 cm × 25.8 cm). CNO was administered by intraperitoneal injection (5 mg/kg) to experimental mice 30 minutes before testing, while the control group was injected with saline 30 minutes before testing. Right, percent distance that mice traveled in the center zone over the total distance they traveled in the open field chamber in 10 minutes (*n* = 11 mice for saline, *n* = 10 mice for CNO treatment). The group average data are represented mean ± SE. Control saline and CNO treated mice do not show significant differences in their traveling distance in the center zone relative to the open field (no significance, n.s., *p* = 0.467, Mann–Whitney U test). **(C)** Schematic illustration of the EPM test and experimental results following the CNO/hM4D-inactivation of dCA3-projecting vCA1 neurons. Left, the EPM apparatus consists of 2 open and 2 closed arms (25 cm × 5 cm) with a height of 50 cm from the ground. Mice were initially placed in the center facing an open arm, and they were allowed to explore for 5 minutes. Middle, the numbers of entries to closed arms, open arms, and center location were measured. They do not differ between control saline and CNO injection (*n* = 10 mice for control, *n* = 9 mice for CNO; closed arm, *p* = 0.701; center, *p* = 0.795; open arm, *p* = 0.921, Mann–Whitney U tests). Right, plots of times that mice spent in each location (n.s., closed arm: *p* = 0.986; center: *p* = 0.983; open arm, *p* = 0.888, Mann–Whitney U tests). The group average data are represented mean ± SE. **(D)** Illustration of the OLM test, and experimental results with the CNO/hM4D-inactivation of dCA3-projecting vCA1 neurons. Left, the box (23 cm × 30 cm) represents the arena for the OLM test with 2 identical objects in the training and testing sessions. The objects (indicated by blue-filled circles) are placed in the arena in the training session, and 24 hours later, one of the 2 objects is moved to a new location in the testing session. The animals were injected with CNO (*n* = 10 mice) or saline (*n* = 11 mice) 30 minutes before the training session and allowed to explore the objects in the box for 10 minutes. Middle, the mice do not differ in their overall DI measurements for the training session (n.s., *p* = 0.905, Mann–Whitney U test). Right, the DI measurements for the testing session at 24 hours after training differ between groups. ***, *p* = 0.0001 (Mann–Whitney U test). The group average data are represented as mean ± SE. **(E)** Illustration of the novel ORM test and experimental results. Two identical objects (indicated by gray-filled circles) are placed in the arena in the training session, and 24 hours later, one of the objects is replaced with a new object indicated by a blue, filled triangle at the same location in the testing session. Middle, the DI measurements for the training session (n.s., *p* = 0.973, Mann–Whitney U test). Right, the DI measurements for the testing session differ significantly between groups. ***, *p* = 0.003 (Mann–Whitney U test). The group average data are represented mean ± SE. The raw data for Fig 7B–7E are included in S3 Data. CNO, clozapine N-oxide; dCA3, dorsal CA3; DI, discrimination index; EPM, elevated plus maze; OLM, object location memory; ORM, object recognition memory; vCA1, ventral CA1.

The ventral hippocampus processes neural circuit information related to emotional memory and anxiety [2,45,46]. To investigate whether the vCA1 to dCA3 pathway modulates anxiety-related behaviors, we used designer receptors exclusively activated by designer drugs (DREADDs) to inactivate dCA3-projecting vCA1 neurons during the mouse behavioral testing in the open field and elevated plus maze (EPM; Fig 7A–7C). These anxiety-related tests are based on the natural aversion of animals to open, novel, and elevated spaces [47]. With the bilateral injection of CAV2-Cre in dCA3 and AAV2-DIO-hM4D-mCherry in vCA1, the hM4D selectively expresses in Cre positive neurons in the pyramidal layer of vCA1 (Fig 7A, S4A Fig) [48]. clozapine N-oxide (CNO, 5mg/kg) or saline is intraperitoneally administrated 30 minutes before behavioral tasks [49].

We first measured the locomotor activities in the open arena. Compared with saline-injected control mice, the CNO-treated group does not display significant differences in total locomotor movements (saline, 1,712.89 ± 131.37 cm, *n* = 11 mice; CNO, 1,734.34 ± 153.86 cm, *n* = 10 mice; *p* > 0.999, Mann–Whitney U test) and the percentage of distance traveled in the center zone (saline, 15.84 ± 2.33%, *n* = 11 mice; CNO, 17.26 ± 1.86%, *n* = 10 mice; *p* = 0.467, Mann–Whitney U test) (Fig 7B, S4B Fig).

In the EPM test, the animals with CNO inhibition of dCA3-projecting vCA1 neurons do not show differences in their numbers of entries to each arm and the center space compared with animals in the saline group (saline treatment, close arm: 14.2 ± 0.96 entries, center: 13.20 ± 1.24 entries, open arm: 2.70 ± 0.68 entries, *n* = 10 mice; CNO treatment, close arm: 14.56 ± 2.12 entries, center: 12.78 ± 1.69 entries, open arm: 2.78 ± 0.80 entries, *n* = 9 mice; close arm: *p* = 0.701; center: *p* = 0.795; open, *p* = 0.921, Mann–Whitney U tests). The time spent in each location is also unaffected by the inhibition of dCA3-projecting vCA1 neurons (saline treatment, close arm: 237.80 ± 13.34 seconds, center: 36.80 ± 6.26 seconds, open arm: 24.40 ± 7.08 seconds, *n* = 10 mice; CNO treatment, close arm: 239.33 ± 12.23 seconds, center: 39 ± 7.21 seconds, open arm: 20.33 ± 6.12 seconds, *n* = 9 mice; close arm: *p* = 0.986; center: *p* = 0.983; open arm, *p* = 0.888, Mann–Whitney U tests) (Fig 7C).

Recent studies report that the ventral hippocampus neurons exhibit broadly tuned place fields, suggesting a role in the spatial memory. Thus, we tested animals in an OLM task. In this task, a training session involving exploration of an environment containing 2 objects is followed a day later by a testing session in the same environment but with alteration of the location of one of the objects. Time spent exploring each object is determined in each session and a discrimination index (DI) comparing time spent at one versus the other object is calculated. Memory for the original location configuration of the 2 objects is measured on the test day as a DI favoring encounter and exploration of the moved object.

We find that hM4D-mediated inhibition of dCA3-projecting vCA1 neurons during the training session significantly lowers the testing day DI compared with control mice administered with saline (saline treatment, 34.29 ± 4.97%, *n* = 11 mice; CNO treatment, −3.59 ± 6.20%, *n* = 10 mice; *p* = 0.0001, Mann–Whitney U test) in the testing session (Fig 7D). Thus, dCA3-projecting vCA1 neurons are functionally implicated in the training day development of OLM.

We also tested animals using an object recognition task in which 1 of 2 objects presented during a training session is replaced by a novel object at the same location prior to the following day’s test session. The testing day DI measures recognition of the replaced object as a bias to explore the newly placed object. We find the DI is much lower in the hM4D/CNO-inactivation group compared with the control group (saline treatment, 27.71 ± 3.47%, *n* = 11 mice; CNO treatment, −3.4 ± 4.27%, *n* = 10 mice; *p* = 0.0003, Mann–Whitney U test) during the testing session (Fig 7E).

We find no differences in the DI between the control and CNO group (Fig 7D and 7E) during the training sessions, indicating balanced encounter and exploration of the 2 objects. Consistent with open field results, CNO does not affect locomotor activity generally as the average total exploration time of the CNO group does not differ from the control group (S4C Fig).

Our behavioral data with genetic inactivation of dCA3-projecting vCA1 neurons support the notion that the vCA1 to dCA3 projection modulates object-related spatial memory but do not modulate anxiety-related behaviors. However, we acknowledge an interpretational caveat regarding the specificity of inactivating vCA1-dCA3 projections in our DREADDs experiments, as dCA3-projecting vCA1 excitatory cells could have multiple axonal collaterals projecting also into other brain areas in parallel with the dCA3.

## Discussion

Using multiple retrograde and anterograde viral tracers, we have identified and quantitatively mapped noncanonical circuit inputs to dCA3. Unexpectedly, we discovered a prominent back projection pathway from vCA1 to dCA3 running opposite the trisynaptic pathway and opposite the septotemporal axis. Furthermore, we find that noncanonical CA3 inputs include those from the subicular complex and Prh. These noncanonical input strengths vary with dCA3 locations along the transverse axis. The more extensive vCA1 to dCA3 pathway specifically modulates spatial and object related memory behaviors but not anxiety-related behaviors. Together, our data support the existence of extensive, noncanonical circuitry in the HF. These noncanonical projections from vCA1, subicular complex and Prh to CA3 may modulate the trisynaptic pathway through the hippocampus in a topographic fashion.

**Fig 8:**
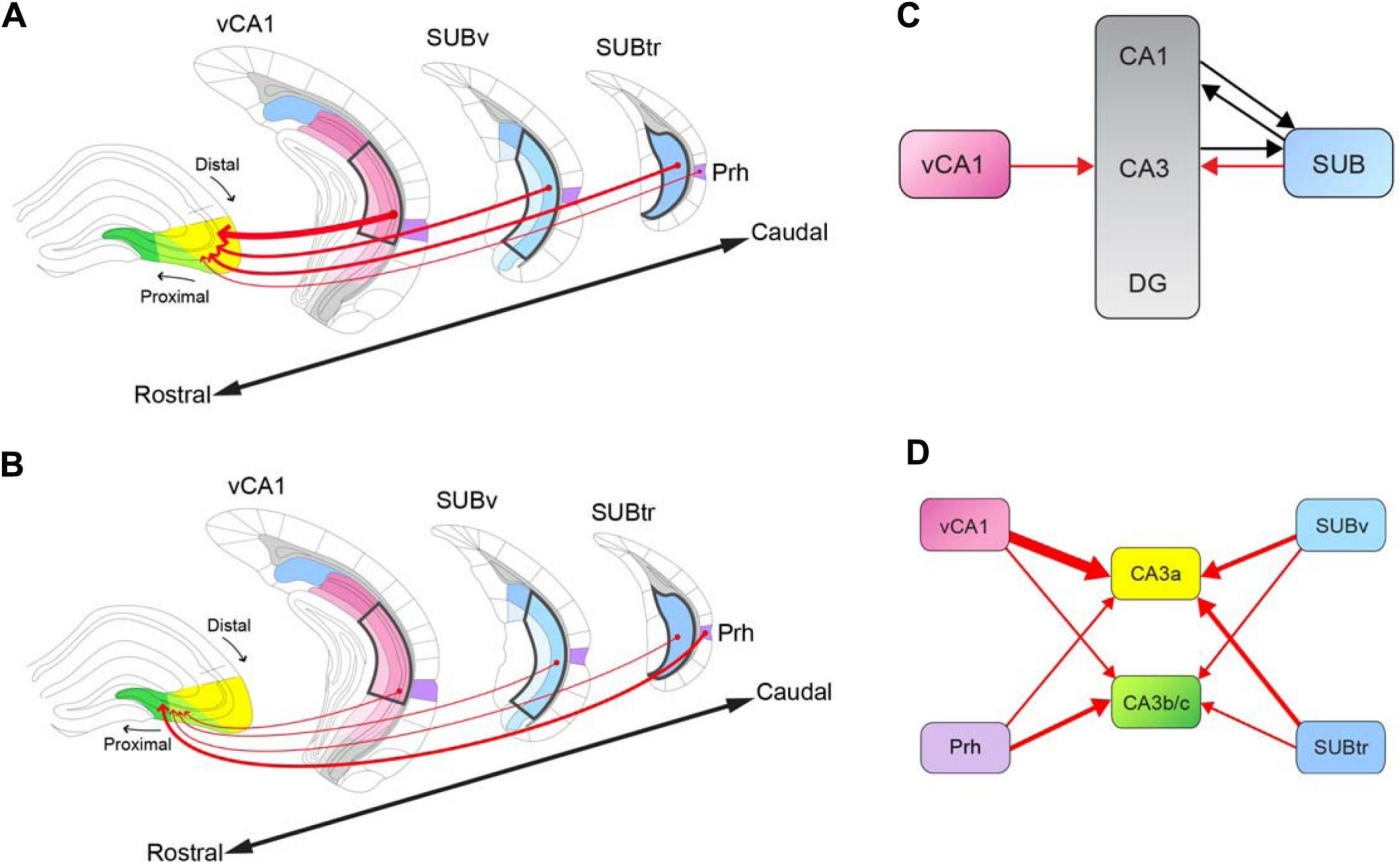
Schematic summary of canonical hippocampal circuitry and the noncanonical inputs from vCA1, SUB complex, and Prh to dCA3 subregions. **(A)** The summary diagram of spatial topology of noncanonical inputs to the more distal region of dorsal CA3 (CA3a). The input regions, vCA1, SUBv, SUBtr, and Prh, are organized from the rostral to caudal direction. Gradient colors of blue (the subicular complex regions) and pink (vCA1 subregions) represent the subregions of each input region. Shades of the same color indicate different layers in each region. The projection direction is indicated by a red arrow, and the thickness of the arrow indicates the connectivity strength. **(B)** The diagram is formatted similarly to A to depict the noncanonical inputs to the more proximal regions of dCA3 (CA3b and CA3c). **(C)** The diagram depicts the novel noncanonical pathways that project in the opposite direction of the trisynaptic pathway. The noncanonical back projections from SUB and vCA1 to CA3 are depicted as red lines with arrows. The previously identified feedforward projections (CA1-SUB) and noncanonical projections (SUB-CA1) [14–16] and CA3—SUB [37] are depicted as black lines with arrows. The vCA1 and SUB label gradients are used to indicate the subregions as matched to the corresponding colored regions in A and B. The SUB gradient is composed of the SUBv and SUBtr subregions. **(D)** The diagram of differential input strengths and patterns to the dCA3 subregions, CA3a and CA3b/c. Color-labeled regions are corresponding to the input regions in A and B. The size of red arrows indicates the connectivity strength. dCA3, dorsal CA3; Prh, perirhinal cortex; SUB, subiculum; SUBtr, subiculum transition area; SUBv, ventral subiculum; vCA1, ventral CA1.

Previous studies have established the basic architecture of HF connectivity, whereas cell type–specific connections and the quantification of connectivity strengths remain less clearly revealed. Leveraging new viral genetic tools, we discovered noncanonical projection pathways which have not been appreciated in previous studies. Specifically, we observed projections from vCA1, Prh, SUBv, and SUBtr to dCA3 excitatory neurons (Fig 8). The connectivity strength of vCA1, SUBv, and SUBtr quantified by the ratio of presynaptic input neurons over the starter neurons in the injection site gradually decreases along the transverse axis from distal CA3 to proximal CA3. The Prh projection to CA3 varies oppositely, increasing in strength at proximal CA3 (Fig 8A and 8B). Rabies viral tracing data displays consistent results with the other 2 retrograde viral tracers, CAV2-Cre, rAAV-retro-Cre, and anterograde viral tracer (H129-G4). Thus, our new viral tracing data demonstrate previously undefined noncanonical pathways and provide alternative perspectives to understand the heterogeneity of CA3 observed in neurophysiological, genomic, and functional studies [22,23,25].

Technical limitations restrict neural circuit mapping applications by CAV2-Cre and rAAV2-retro-Cre. These viral labeling methods are useful for qualitative assessments but not for strict quantification of input strengths compared with genetically modified rabies viral tracing, as they do not provide assessments of the numbers of “starter cells.” In comparison, monosynaptic retrograde rabies tracing is amenable to semiquantitative analysis. We are able to quantitatively analyze the strength of input connection (CSI) because of the feature of helper AAV and rabies virus expressing measurable numbers of “starter cells” in the injection site. We determined the quantitative CSIs of CA3 inputs using rabies virus tracing, and we find the strength of inputs follow a gradient pattern along the CA3 transverse axis. While we are not able to compare the semiquantitative strength of inputs to CA3 subregions in the CAV2-Cre and AAV2-retro cases, these methods provide qualitative support for our overall findings and, in particular, the presence of projection pathways from vCA1, ventral SUB, and Prh to dCA3. Note that the total cell counts for rabies tracing cases are overall lower than CAV2-Cre and rAAV2-retro-Cre cases (S4 Table). We do not expect the rabies or other methods to label every input to each neuron, but this limitation does not mean that our rabies method is not effective. Our earlier study indicates that our rabies tracing system works in a nonbiased fashion [13]. The method of rabies labeling is reliable, because labeled cells are seen in brain structures such as MS-DBB, DG, EC, and MnR that are known to project to hippocampal CA3.

Our study suggests that CAV2-Cre and rAAV-retro-Cre could be used as tools to manipulate the excitatory projection neuron in specific brain regions. The CAV2-Cre virus labels around 98% of excitatory neurons that are CaMKIIα immunopositive in the input mapped regions. We used CAV2-Cre in combination with local AAV-mediated Cre-dependent expression of DREADDs to inhibit vCA1 excitatory projection neurons in the C57 mice. There are heterogeneous groups of interneurons present in all the layers of CA3 and other subfields of the hippocampus. Many types of interneurons including basket and chandelier cells, in the subregions of CA3 receive all major sources of excitatory afferents [50,51]. For example, CA3 interneurons are involved in a feedforward inhibition circuitry activated by dentate mossy fibers [52,53]. Also, an inhibitory feedback pathway from the CA1 area to CA3 and hilar regions has been reported [54]. In this context, it is relevant that our anterograde tracing results show that noncanonical presynaptic inputs may innervate CA3 GABAergic neurons as well as excitatory cells (Fig 6D).

We observed inputs to dCA3 from 2 subregions of the subicular complex: the designated SUBv and the SUBtr. The SUBv has widespread projections to brain regions such as the lateral septum, amygdala, bed nucleus of the stria terminalis, hypothalamus, and lateral EC; these regions are implicated in reward, emotion, stress, and motivation [37]. The SUBtr topographically projects to the retrosplenial cortex, PaS, PrS, medial EC, medial mammillary nucleus, and the anteroventral nucleus of the thalamus; these brain structures are implicated in the encoding of spatial location and head orientation [55–58]. Both subregions of the subicular complex project more heavily to the distal portion of dCA3, overlapping with the more extensive medial EC as opposed to lateral EC inputs to dCA3. This implies a role for this projection in the encoding of location and orientation, but evidence for this awaits detailed electrophysiological studies of SUB neurons in behaving animals.

The discovery of the noncanonical pathways from vCA1i, SUBv, and SUBtr to dCA3 extends the knowledge of HF connectivity and its relation to learning and memory processes across the septotemporal axis. Our previous studies have shown a dorsal SUB back projection to the dorsal CA1 excitatory neurons that plays an important role in facilitating OLM [12]. The present study reveals significant back projections from vCA1 and SUB to the excitatory neurons spatially located in distal CA3. Together, these noncanonical pathways, running in the opposite direction of the traditional trisynaptic pathway, were mapped from SUB to CA1 and from CA1 to CA3. These noncanonical projections, therefore, appear to complement and augment the trisynaptic pathway within the HF.

Our tracing data also support a new way of considering how the hippocampus processes spatial information along the septotemporal axis. Numerous lesion studies have shown that damage to the dorsal hippocampus impairs spatial memory, while animals with damage to the ventral hippocampus display deficits in emotional memory [45,59,60]. Recent physiological studies have observed theta oscillations traveling from the dorsal to the ventral hippocampus [61,62]. This implies an information processing scheme by which information from the dorsal hippocampus is integrated into the ventral regions, finally forming an output to regions such as the prefrontal cortex. In support of this, it is known that both dorsal and ventral hippocampus can represent the location of the animal in the environment but at increasingly large scales of representation in the ventral hippocampus [63,64]. However, the anatomical connections and functional comparison along the hippocampal septotemporal axis are still in question. Our noncanonical vCA1-dCA3, SUBv-dCA3, and SUBtr-dCA3 pathways provide evidence for direct synaptic connections between dorsal and ventral HF, which are opposite to the direction of the trisynaptic pathway and opposite to the flow direction of theta oscillations from septal to temporal poles.

Our behavioral data provide evidence that the vCA1 to dCA3 pathway along the longitudinal axis is critical for object and spatial memory rather than emotional processes. Both object recognition and OLM development during training sessions are impacted by inhibition of vCA1 neurons projecting to dCA3. Kesner’s group observed that lesion damage of vCA1 results in a mild deficit in the temporal ordering of visual objects [65]. Another study used the neuronal activity marker Arc to demonstrate that vCA1 and CA3 are involved in both spatial and nonspatial recognition memories [66]. Our novel object testing result is consistent with these studies and provides convincing evidence of the functional roles of the hippocampus along the longitudinal axis. In future work, it will be of interest to examine how the other noncanonical inputs including subicular complex and Prh, converge in the dCA3 and modulate learning and memory, and object/location representation by dCA3 neural ensembles.

## Materials and methods

### Animals

All experiments were conducted according to the National Institutes of Health guidelines for animal care and use and were approved by the Institutional Animal Care and Use Committee (IACUC) and the Institutional Biosafety Committee of the University of California, Irvine (IACUC protocol #: AUP-20-002). In the viral circuit tracing experiments, Ai9 Cre-reporter mice were used to study CA3 circuit connections. Transgenic Camk2α-Cre and CaMK2α-Cre; TVA mice were used to map the input connections of CA3 excitatory cells with genetically modified rabies virus. Ai9, Camk2α-Cre, and TVA mice have the same C57BL/6 genetic background. Wild-type C57BL/6J mice were used to verify the noncanonical projections of input mapped brain regions with the anterograde herpes virus (H129-G4). At least 60 mice were used for the experiments, with detailed quantification performed in 35 high-quality cases. Another cohort of C57BL/6J mice was used to examine the functional roles of dCA3-projecting vCA1 neurons in the behavioral tests. See the text in S1 Table for detailed information.

### Viral injections

Viral injection procedure follows a previously described protocol [13]. Mice were anesthetized under 1.5% isoflurane for 10 minutes with a 0.8 L/min oxygen flow rate using an isoflurane tabletop unit (HME109, Highland Medical Equipment, Temecula, CA, USA). Mice were transferred to a rodent stereotaxic frame (Leica Angle Two for mouse, Leica Biosystems Inc., Buffalo Grove, IL, USA), and they were anesthetized with a continuous 1% flow of isoflurane. A small incision was made in the head to reflect the skin, and the skull was exposed to show the landmarks of bregma and lambda. A 3-axis micromanipulator guided by a digital atlas was used to calculate the coordinates of the injection site relative to the bregma and lambda. The virus was delivered to the target region. We introduced 2 delivery methods for viral tracers: picospritzer pressure injection and iontophoretic current injection. Pressure injection infects a large number of cells in the injection site, but current injection restricts the size of infected areas within a small target region. Both injection methods show no biased inputs from different brain regions. For pressure injection, a small drill hole was made in the skull above the injection site, exposing the pia surface. A glass pipette (tip diameter, approximately 20 to 30 μm) was loaded with the virus and then lowered into the brain at appropriate coordinates. A picospritzer (Parker Hannifin, Hollis, NH, USA) was used to pulse the virus into the brain at a rate of 20 to 30 nl/min with a 10-ms pulse duration. For iontophoresis, the virus was delivered with a positive 3-μA current in a cycle of 7 seconds “on” and 7 seconds “off” for a duration of 10 minutes. The injection pipette was remained in the brain for 5 minutes after completion of the injection to prevent backflow of the virus. Once the injection pipette was withdrawn, the mouse was removed from the stereotaxic frame, and the incision was closed with tissue adhesive (3M Vetbond, St. Paul, Minnesota, USA). Mice were given an injection of Carprofen and taken back to recover in their home cages.

### CAV2-Cre virus

To study the circuit input connections of CA3 neurons, 0.2 μl of CAV2- ΔE1-Cre virus (2.6 × 10^12^ infectious units per ml, purchased from E.J. Kremer’s group, France) was injected into the dCA3 of each Ai9 mouse. After 3 weeks, the Ai9 mice were perfused for tissue processing.

### rAAV2-retro-Cre virus

To confirm the CAV2-Cre results, we used the rAAV2-retro-Cre virus in the viral tracing experiments. Moreover, 0.1 μl of rAAV2-retro-hSyn-Cre virus (1.57 × 10^13^ genomic units per ml, custom packaged by Vigene Biosciences, Rockville, MD, USA) was injected into the dCA3 of each Ai9 mouse. After 3 weeks, the Ai9 mice were perfused for tissue processing.

### Helper AAV and rabies viruses

To map and quantitatively analyze the input strengths of excitatory CA3 cells in the CA3 subregions, we used genetically modified rabies virus and transgenic mouse lines expressing Cre in CaMKIIα-expressing excitatory neurons. Rabies virus was made locally at the Center for Neural Circuit Mapping Center of the University of California, Irvine, with required cell lines and seeding viruses originally from E. Callaway’s group at the Salk Institute for Biological Studies. The helper AAV8-EF1a-DIO-H2B-GFP-2A-OG (1.54 × 10^13^ genome units per ml, custom packaged by Vigene Biosciences, Addgene plasmid #74289) was delivered through iontophoresis into target subregions in double transgenic mice of Camk2α-Cre; TVA. In separate experiments, the helper AAV8-hSyn-DIO-TC66T-2A-eGFP-2A-OG (1.8 × 10^12^ genome units per ml; custom packaged by Vigene Biosciences) was delivered into the CA3 subregions of the Camk2α-Cre mouse line. The coordinates of dCA3 subregions relative to the bregma are AP: −1.94mm, mediolateral (ML): −2.48 mm, dorsoventral (DV): −2.24 mm for CA3a, AP −1.94 mm, ML: −1.97 mm, DV: −2.24 mm for CA3b, and AP: −2.06mm, ML: −1.92 mm, DV: −2.13 mm for CA3c (Fig 4B–4D, S1 Table). Three weeks after the AAV injection, which allows for the infected neurons to express high levels of RG and EGFP, the pseudotyped G-deleted rabies virus (EnvA-SADΔG-RV-DsRed, 0.4 μl, approximately 2 × 10^7^ infectious units per ml) was injected into the same target region as the helper AAV. The rabies virus was allowed to replicate and retrogradely spread from targeted Cre+ cell types to directly connected presynaptic cells for 9 days before the mice were perfused for tissue processing.

### H129-G4 virus

To examine the projections of input mapped brain regions, 0.1 ul of anterograde-directed herpes virus (H129-G4, 2.35 × 10^7^ infectious units per ml, original reagents from the Luo Lab (Wuhan, China); produced locally at the Center for Neural Circuit Mapping Center, Irvine, CA, USA) was injected at the following coordinates relative to the bregma: AP: −3.28 mm, ML: −3.5 mm, DV: −3.11 mm for vCA1; AP: −4.16 mm, ML: −3.22 mm, DV: −3.45 mm for SUBv. Red Retrobeads (Retrobeads IX, Lumafluor, Durham, CA, US) were mixed with H129-G4 (ratio 1:1) to facilitate the identification of the hits of a target region. H129-G4 virus was allowed to replicate and anterogradely spread to postsynaptic neurons for 48 hours before the animals were perfused for tissue processing. We only used verified cases in which the red Retrobeads were restricted to the injection site, either vCA1 or SUBv. The cases with leakage to the surrounding brain regions such as DG, CA3, and PaS were excluded.

### AAV2-DIO-hM4D-mCherry

For the genetic inactivation of dCA3-projecting vCA1 neurons, wild-type C57BL/6J mice were injected with CAV2-Cre at bilateral CA3 (ML: ±2.48 mm; AP: −1.94 mm; DV: −2.24 mm). Then, 0.3 μl of AAV2-DIO-hM4D-mCherry (3.7 × 10^12^ genomic copies/ml; UNC Vector Core) were delivered to vCA1 bilaterally (ML: ±3.5 mm; AP: −3.28 mm; DV: −3.11 mm) in the same mouse. Mice were then allowed to recover in their home cages for 3 weeks before behavior experiments.

### Histology and immunochemical staining

The mice were perfused with 5 ml of PBS, followed by 25 ml PBS containing 4% paraformaldehyde. The perfused mice brains were post fixed in 4% paraformaldehyde and were switched into 30% sucrose in 1 X PBS 24 hours later. The brain was frozen using dry ice and coronally sectioned in 30-μm thickness on a microtome (Leica SM2010R, Germany). One out of every 3 sections was mounted for examination of virally labeled neurons in different brain structures. These sections were imaged for all subsequent computer-based analyses. Some of the remaining sections were selected for neurochemical characterization of labeled cells. To identify the neurochemical cell types of CAV2-labeled presynaptic neurons, GABAergic and CaMKIIα, immunostaining was performed. For GABA staining, a rabbit anti-GABA primary antibody (Sigma-Aldrich (St. Louis, MO, USA), A2052, 1:4,000 dilution) was used followed by an Alexa Fluor (AF) 488- or Cy5-conjugated donkey anti-rabbit secondary antibody (Jackson ImmunoResearch (West Grove, PA, USA), 1:200 dilution). To examine excitatory cell labeling, selected sections were immunolabeled by a mouse anti-CaMKIIα primary antibody (Thermo Fisher Scientific (Riverside, CA, USA), MA1-048, 1:100 dilution) followed by the AF488- or Cy5-conjugated donkey anti-mouse secondary antibody. To delineate hippocampal CA2 region, selected hippocampal sections were stained with a rabbit PCP4 antibody (Invitrogen (Carlsbad, CA, USA), PA5-52209, 1:1000) followed with the Cy5-conjugated donkey anti-rabbit secondary antibody (Jackson ImmunoResearch, 1:200 dilution).

### Data quantification

Brain slice images were acquired by using an automated slide scanning acquisition software (Metamorph, MDS Analytical Technologies, Sunnyvale, CA, USA) in a high-capacity computer coupled with a fluorescent BX61 Olympus microscope and a high-sensitivity Hamamatsu CCD camera. In addition, we imaged labeled cells in selected sections with a confocal microscope (LSM 700/780, Carl Zeiss Microscopy, Nussloch, Germany) coupled with z-stack and tile scanning features under 20X, 40X, and 63X objectives. The confocal imaging system uses dichroic mirrors for multicolor imaging. Typically during 20x imaging, for data acquisition by 633-nm excitation, the emission signal was acquired from 652 nm to 728 nm (pinhole: 131 μm). For data acquisition by 561-nm excitation, the emission was acquired from 588 nm to 650 nm (pinhole: 46 μm). For data acquisition by 488-nm excitation, the emission was acquired from 490 nm to 552 nm (pinhole: 81 μm). For data acquisition by 405-nm excitation, the emission was acquired from 409 nm to 489 nm (pinhole: 46 μm). The image stack (about 20-μm thick) was acquired by the Zen software using the Carl Zeiss Image Data File format (.CZI), then converted to a maximal projection image in a TIFF format. Quantitative examinations across the series of sections were conducted for complete and unbiased analyses of virally labeled neurons by using Adobe Photoshop software (Adobe Systems, San Jose, California, USA).

For rabies tracing experiments, we followed the established counting protocol in the previous publication [13]. We first selected the brain section with the target region, dCA3 to identify EGFP and DsRed doubled-labeled starter neurons that are restricted to CA3. All the starter cells were manually counted using the counting tool in Photoshop. Next, we aligned the rest of the viral-infected brain sections with Franklin and Paxinos’ mouse brain atlas images [34] to determine the anatomical structures for the quantification of labeled cells in specified brain regions. No stereological protocol was used; all labeled cells in each section of the brain section series (i.e., 1 out of every 3 sections was mounted for examination of virally labeled neurons in different brain structures) were counted. An input CSI was operationally defined as the ratio of the number of presynaptic neurons in a brain region of interest (e.g., SUB) versus the number of postsynaptic (starter) neurons in CA3. We also calculated the PI as the ratio of the number of presynaptic inputs versus the number of total inputs. The resulting CSI and PI show similar trends for the overall input regions. For CAV2-Cre and rAAV-retro-Cre tracing experiments, we counted the presynaptic input neurons across the brain section series. We calculated the PI measurement as the ratio of the number of presynaptic inputs versus the number of total inputs.

### Behavioral experiments

#### Drug

CNO (Enzo Life Sciences, Farmingdale, New York, USA; BML-NS105-0025) was dissolved in saline to make a concentration of 1 mg per ml on an experimental day [49]. All the animals were administrated with either CNO or saline intraperitoneally (i.p.) at 5 mg per kg 30 minutes before anxiety-like tests or the training session of the memory tests.

#### Behavioral tasks

All mice were well handled for 7 days and habituated to i.p. injections before experiments. Animals were allowed to acclimate to the testing room in their home cages for 1 hour before placement in the testing apparatus. The anxiety-like tests and the memory tests followed the previous protocols with minor modifications [12,47].

#### Open field

The locomotion test was recorded in a square plexiglass test chamber (40 cm × 40 cm × 50 cm) by an automated video system (Video-Mot II, TSE, Bad Homburg, Germany) [47]. The distance traveled in 10 minutes was measured. The center zone was defined as a 25.8 cm × 25.8 cm square in the middle of the box.

#### EPM

The test was performed in a plus-maze consisted of 2 open (25 cm × 5 cm) and closed arms (25 cm × 5 cm × 15 cm) connected by a central platform (5 cm × 5 cm). The maze was 50 cm in height from the ground. Mice were released at the central platform facing an open arm. The numbers of entries and the time spent in each arm and the center zone were measured over 5 minutes of exploration time. The entry was defined as all 4 paws entering a new compartment.

#### OLM test

Animals were habituated in a rectangle arena (23 cm × 30 cm) for 7 days before the training session. The walls of the arena were adorned with fixed visual cues. In the training session, 2 identical objects were placed in the box and mice had 10 minutes of free exploration. One of the objects was moved to a new location on the testing day 24 hours after the training session. The mice were allowed to freely explore in the box for 5 minutes.

#### ORM test

The ORM test was performed similarly to the OLM test, except for the testing session. In this session, instead of changing the location of one of the original objects, the object was replaced with a novel object. Different sets of objects were used in OLM and ORM tests.

Only mice with sufficient object encounter and exploration (>3 seconds) in both training and testing sessions were included for analysis. The behavioral data were analyzed following the criteria in the previous publications [12]. The exploration time is defined as the time when the mouse’s nose is within 1 cm of the object and/or sniff the object. The following behaviors do not count as exploring: (1) the animal is not approaching to the object (e.g., the nose comes close to the object during reorientation); (2) the animal mounts/rears on the top of the object (e.g., looking at the ceiling); and (3) the animal engages in repetitive behavior (such as digging close to the object or biting the object). For the testing sessions, the DI is defined as (T_moved_−T_unmoved_)/(T_moved_ + T_unmoved_) × 100% or (T_novel_ − T_familiar_)/(T_novel_ + T_familiar_) × 100%. T_moved_ and T_unmoved_ refer to the time spent exploring the unmoved and moved objects, respectively, and T_novel_ and T_familiar_ refer to the time spent exploring the novel and familiar objects, respectively.

### Statistics

Data are presented as the mean ± SE. We applied appropriate statistical tests, and the data analysis was conducted using GraphPad Prism (GraphPad software, San Diego, CA, USA). For statistical comparisons across more than 2 groups, the nonparametric version of 1-way ANOVA (Kruskal–Wallis test) was first used, and if the outcome was significant, Dunn multiple comparison tests were conducted between groups with multiple comparison corrections as needed. For statistical comparisons between groups, a Mann–Whitney U test was used. This is in line with routine guidelines for a relatively small sample size and does not require assumptions of normality or equal variance required for parametric tests. Alpha levels of *p* ≤ 0.05 were considered significant.

## Supporting information

S1 Fig CAV2-Cre infected excitatory neurons in the dCA3 injection site and the presynaptic input regions. **(A)** Example images of CaMKIIα and GABA dual immunostaining of CAV2-labeled presynaptic neurons in dCA3. CAV2-Cre infected neurons are labeled by tdTomato in the Ai9 mouse, CaMKIIα immunoreactivity is visualized with an AF488-conjugated secondary antibody, and GABA immunoreactivity is revealed with a Cy5-conjugated secondary antibody and presented as a blue pseudo-color. The arrow in A indicates one CAV2-labeled cell that is positive for GABA immunostaining. The arrowhead points to a GABAergic cell that is not CAV2-Cre labeled. **(B-D)** Examples of immunostaining results in vCA1, SUBtr, and Prh, following the same format as in A. The scale bar (20 μm) applies to all the panels. CaMKIIα, calmodulin-dependent protein kinase IIα; dCA3, dorsal CA3; Or, oriens cell layer; Prh, perirhinal cortex; Py, pyramidal cell layer; SLu, stratum lucidum of the hippocampus; SUBtr, subiculum transition area; vCA1, ventral CA1.
(PDF)

S2 Fig Unambiguous identification of starter cells and quantification of starter cells in the injection sites. **(A–H)** Example section images show the distribution of starter neurons in the CA3 injection region. The expression of rabies virus (EnvA-SADΔG-RV-DsRed) is visualized with DsRed, the expression the helper AAV (AAV8-hSyn-DIO-TC66T-2A-eGFP-2A-OG) is visualized with EGFP. DAPI staining is blue. The starter cells can be unambiguously identified by their EGFP and DsRed expression from both the helper AAV and ΔG-DsRed rabies virus. The number of starter neurons in each brain section is 4, 6, 7, 4, 1 and 0 for A, B, C, E, F, and H, respectively. The AP number indicates the distance from the coronal section (30-um thick) to the bregma. D and G are the enlarged confocal images of the white boxed regions in panels C and F. The white arrows point to all the starter neurons in the coronal sections. **(I)** Example images of helper AAV (AAV-DIO-H2B-GFP-2A-OG) infected cells in the CA3 injection site using Camk2a-Cre; TVA mice. The helper virus labels the cells with nuclear localized EGFP. Rabies-infected neurons express DsRed. The white arrows point to the colocalization of AAV-EGFP and rabies infected neurons (starter neurons). DAPI staining is blue. **(J)** Example images of helper AAV (AAV8-hSyn-DIO-TC66T-2A-eGFP-2A-oG) infected cells in the CA3 injection site using Camk2a-Cre mice. **(K)** The average proportion of CA3 starter neurons in the AAV-GFP labeled neurons with different helper AAVs. *n* = 3 mice per helper AAV. No significance is found between 2 helper viruses (*p* = 0.7, Mann–Whitney U test). Data are represented mean ± SE. The raw data for S2K Fig are included in S4 Data. AP, anterior–posterior.
(PDF)

S3 Fig Delineation of hippocampal CA2 with PCP4 immunostaining in virally labeled coronal brain sections. **(A)** From the left to right, the first panel shows a low magnification image of PCP4 staining. The second, third, and fourth panels are the enlarged views of the white boxed region in the first panel. The CAV2-Cre cells are visualized with tdTomato in the Ai9 mouse, PCP4 immunoreactivity is visualized with a Cy5-conjugated secondary antibody but presented as a green pseudo-color. DAPI staining is blue. The star and dashed line indicate the border between distal CA3 and CA2. The arrow in the fifth panel indicates the virally labeled neuron located in CA2. The solid white line indicates the CA2/CA1 border. **(B–E)** Example results of PCP4 immunostaining of more virally labeled brain sections, CAV2-Cre (B), rAAV-retro-Cre (C), rabies virus (D), and H129-G4 (E), following the same format as in A. Rabies-infected neurons are labeled with DsRed in D. H129-G4 infected neurons are visualized with EGFP in E. PCP4 immunoreactivity is revealed with a Cy5-conjugated secondary antibody but presented as a red pseudo-color in E. The scale bar (200 μm) applies to low magnification images in A–E; the scale bar (50 μm) applies to all the high magnification images in A–E.
(PDF)

S4 Fig Histological verification and supporting data for behavioral experiments. **(A)** Top: Example section image of hM4D-mCherry expression in vCA1, following the injection of CAV2-Cre in dCA3 and local vCA1 injection of AAV2-DIO-hM4D-mCherry. Bottom: The high magnification image of a portion of vCA1 shows that AAV-hM4D-infected neurons are localized in the pyramidal layer. DAPI staining is blue. **(B)** Locomotor activity in the open field box. The total distance traveled in the open arena in 10 minutes is presented (*n* = 11 mice for saline control, *n* = 10 mice for CNO treatment). The group average data are represented mean ± SE. Control and CNO treated mice did not show significant differences in their total traveling distance in the open field (no significance, n.s., *p* > 0.999, Mann–Whitney U test). **(C)** Exploration times for the 2 objects during the training session in OLM and ORM tests. Left, the total exploration time spent at object 1 and object 2 in the OLM training session (no significance, n.s., *p* = 0.917, Mann–Whitney U test). Right, the total exploration time spent at object 1 and object 2 in the ORM training session (no significance, n.s., *p* = 0.972, Mann–Whitney U test). *n* = 11 mice for saline control, *n* = 10 mice for CNO treatment. The raw data for S4B and S4C Fig are included in S3 Data. CNO, clozapine N-oxide; OLM, object location memory; or, oriens layer; ORM, object recognition memory; py, pyramidal layer; vCA1, ventral CA1.
(PDF)

S5 Fig Anatomical delineations of vCA1, SUBv, and SUBtr in coronal brain sections. **(A)** Schematic illustrations of vCA1 at different AP locations. The red arrowheads divide vCA1 into 3 subdivisions, dorsal (vCA1d), intermediate (vCA1i), and ventral (vCA1v). The blue arrowhead indicates the ventral edge of the DG lateral blade and the dorsal edge of the rhinal fissure (rh). The dashed lines indicate the boundaries between these subdivisions. The pink label shows the pyramidal layer of vCA1. **(B)** and **(C)** show delineations of the subregions of SUB complex (SUBdd, SUBv, SUBvv, and SUBtr). AP, anterior–posterior; DG, dentate gyrus; Pas, parasubiculum; Prs, presubiculum; SUB, subiculum; SUBtr, subiculum transition area; SUBv, ventral subiculum.
(PDF)

S1 Table Mouse strains and viral injections. (PDF)

S2 Table Quantitative input strengths of Camk2α-Cre excitatory neurons in the CA3 subregions. (PDF)

S3 Table Statistical comparisons of input strengths to excitatory neurons in CA3 subregions. (PDF)

S4 Table The numbers of labeled neurons in CA3 input mapped regions. (PDF)

S1 Data Underlying data for Fig 3G. (XLSX)

S2 Data Underlying data for Figs 4H1, 4H2, 5G and 5H. (XLSX)

S3 Data Underlying data for S4B and S4C Fig and Fig 7B–7E. (XLSX)

S4 Data Underlying data for S2K Fig. (XLSX)

## Abbreviations

AF: Alexa Fluor
AP: anterior–posterior
CaMKIIα: calmodulin-dependent protein kinase IIα
CAV2: canine adenovirus type 2
CNO: clozapine N-oxide
CSI: connection strength index
dCA3: dorsal CA3
DG: dentate gyrus
DI: discrimination index
DREADD: designer receptors exclusively activated by designer drug
EC: entorhinal cortex
EPM: elevated plus maze
HF: hippocampal formation
HSV: herpes simplex virus
IACUC: Institutional Animal Care and Use Committee
i.p.: intraperitoneally
LEC: lateral entorhinal cortex
MEC: medial entorhinal cortex
MnR: median raphe nucleus
MS-DBB: medial septum and diagonal band of Broca
OLM: object location memory
PaS: parasubiculum
PCP4: Purkinje cell protein 4
PI: proportion of inputs
Prh: perirhinal cortex
PrS: presubiculum
RG: rabies glycoprotein
RM: retromammillary
SUB: subiculum
SUBtr: subiculum transition area
SUBv: ventral subiculum
TVA: tumor virus receptor A
vCA1: ventral CA1
