# Supplementary figures and images for: Noncanonical projections to the hippocampal CA3 regulate spatial learning and memory by modulating the feedforward hippocampal trisynaptic pathway
Source: PLoS Biol. 2021 Dec 20;19(12):e3001127. doi: 10.1371/journal.pbio.3001127 (PMC8741299; doi:10.1371/journal.pbio.3001127)

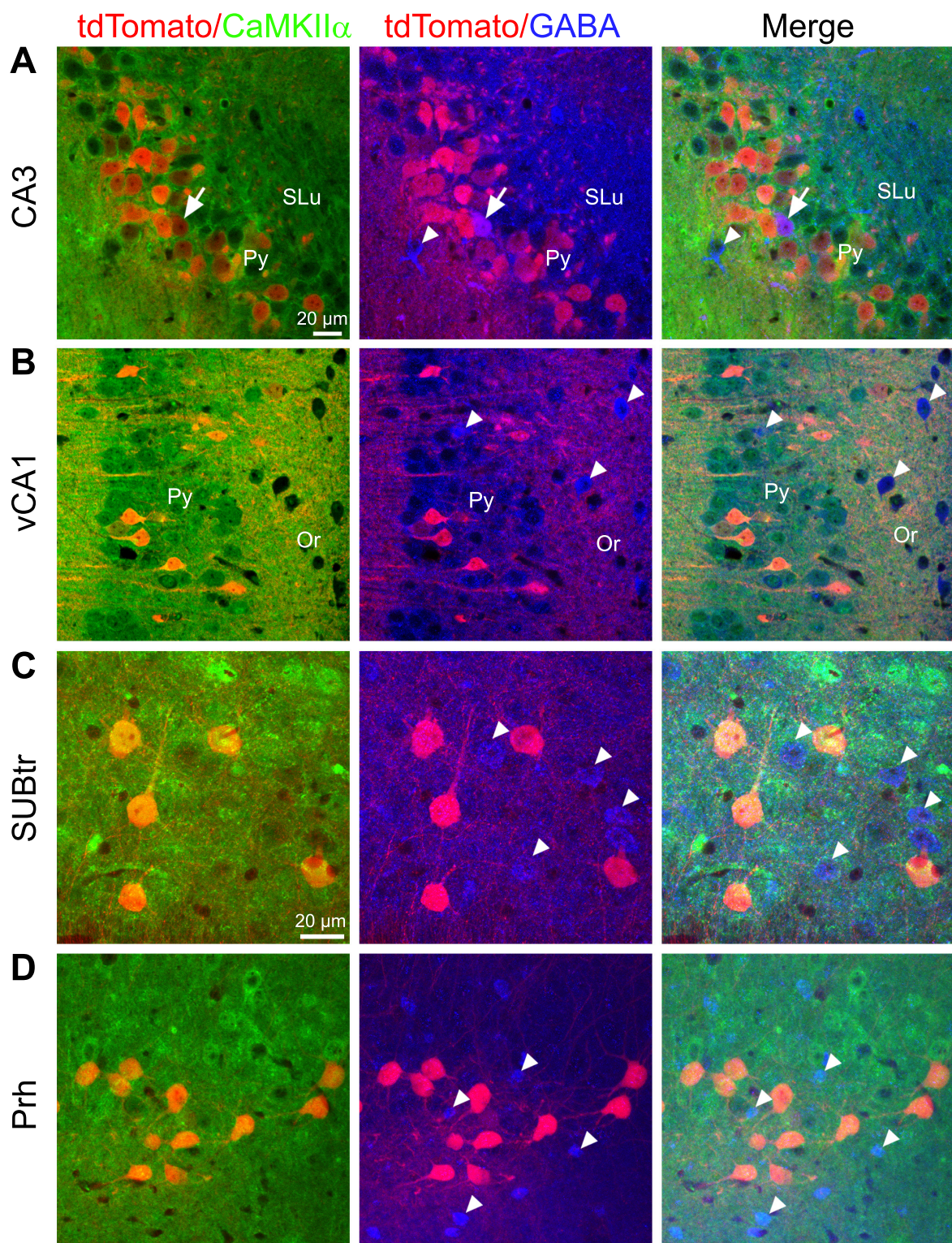

Supplement: S1 Fig — (A) Example images of CaMKIIα and GABA dual immunostaining of CAV2-labeled presynaptic neurons in dCA3. CAV2-Cre infected neurons are labeled by tdTomato in the Ai9 mouse, CaMKIIα immunoreactivity is visualized with an AF488-conjugated secondary antibody, and GABA immunoreactivity is revealed with a Cy5-conjugated secondary antibody and presented as a blue pseudo-color. The arrow in A indicates one CAV2-labeled cell that is positive for GABA immunostaining. The arrowhead points to a GABAergic cell that is not CAV2-Cre labeled. (B-D) Examples of immunostaining results in vCA1, SUBtr, and Prh, following the same format as in A. The scale bar (20 μm) applies to all the panels. CaMKIIα, calmodulin-dependent protein kinase IIα; dCA3, dorsal CA3; Or, oriens cell layer; Prh, perirhinal cortex; Py, pyramidal cell layer; SLu, stratum lucidum of the hippocampus; SUBtr, subiculum transition area; vCA1, ventral CA1. (PDF) [file pbio.3001127.s001.pdf]

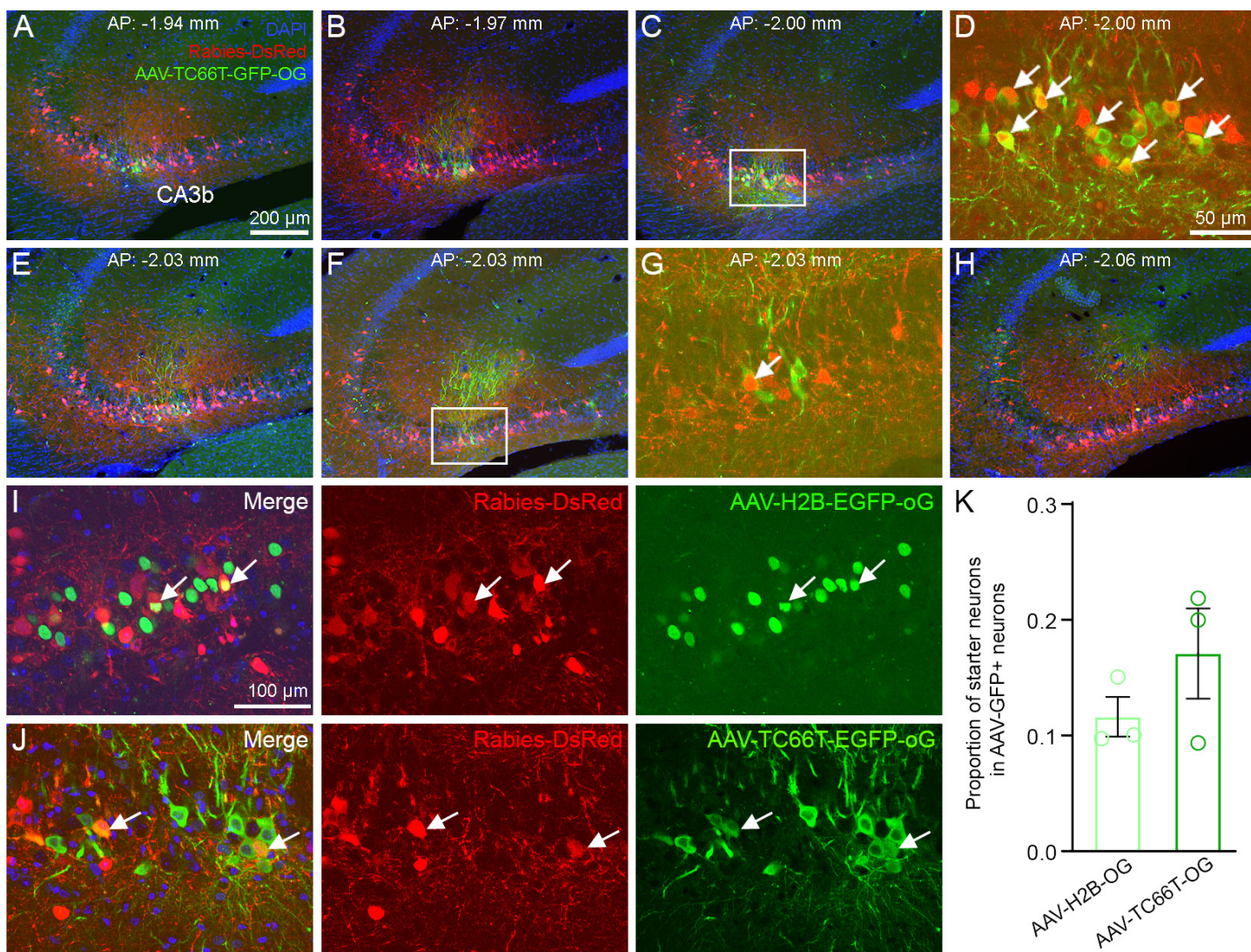

Supplement: S2 Fig — (A–H) Example section images show the distribution of starter neurons in the CA3 injection region. The expression of rabies virus (EnvA-SADΔG-RV-DsRed) is visualized with DsRed, the expression the helper AAV (AAV8-hSyn-DIO-TC66T-2A-eGFP-2A-OG) is visualized with EGFP. DAPI staining is blue. The starter cells can be unambiguously identified by their EGFP and DsRed expression from both the helper AAV and ΔG-DsRed rabies virus. The number of starter neurons in each brain section is 4, 6, 7, 4, 1 and 0 for A, B, C, E, F, and H, respectively. The AP number indicates the distance from the coronal section (30-um thick) to the bregma. D and G are the enlarged confocal images of the white boxed regions in panels C and F. The white arrows point to all the starter neurons in the coronal sections. (I) Example images of helper AAV (AAV-DIO-H2B-GFP-2A-OG) infected cells in the CA3 injection site using Camk2a-Cre; TVA mice. The helper virus labels the cells with nuclear localized EGFP. Rabies-infected neurons express DsRed. The white arrows point to the colocalization of AAV-EGFP and rabies infected neurons (starter neurons). DAPI staining is blue. (J) Example images of helper AAV (AAV8-hSyn-DIO-TC66T-2A-eGFP-2A-oG) infected cells in the CA3 injection site using Camk2a-Cre mice. (K) The average proportion of CA3 starter neurons in the AAV-GFP labeled neurons with different helper AAVs. n = 3 mice per helper AAV. No significance is found between 2 helper viruses (p = 0.7, Mann–Whitney U test). Data are represented mean ± SE. The raw data for S2K Fig are included in S4 Data. AP, anterior–posterior. (PDF) [file pbio.3001127.s002.pdf]

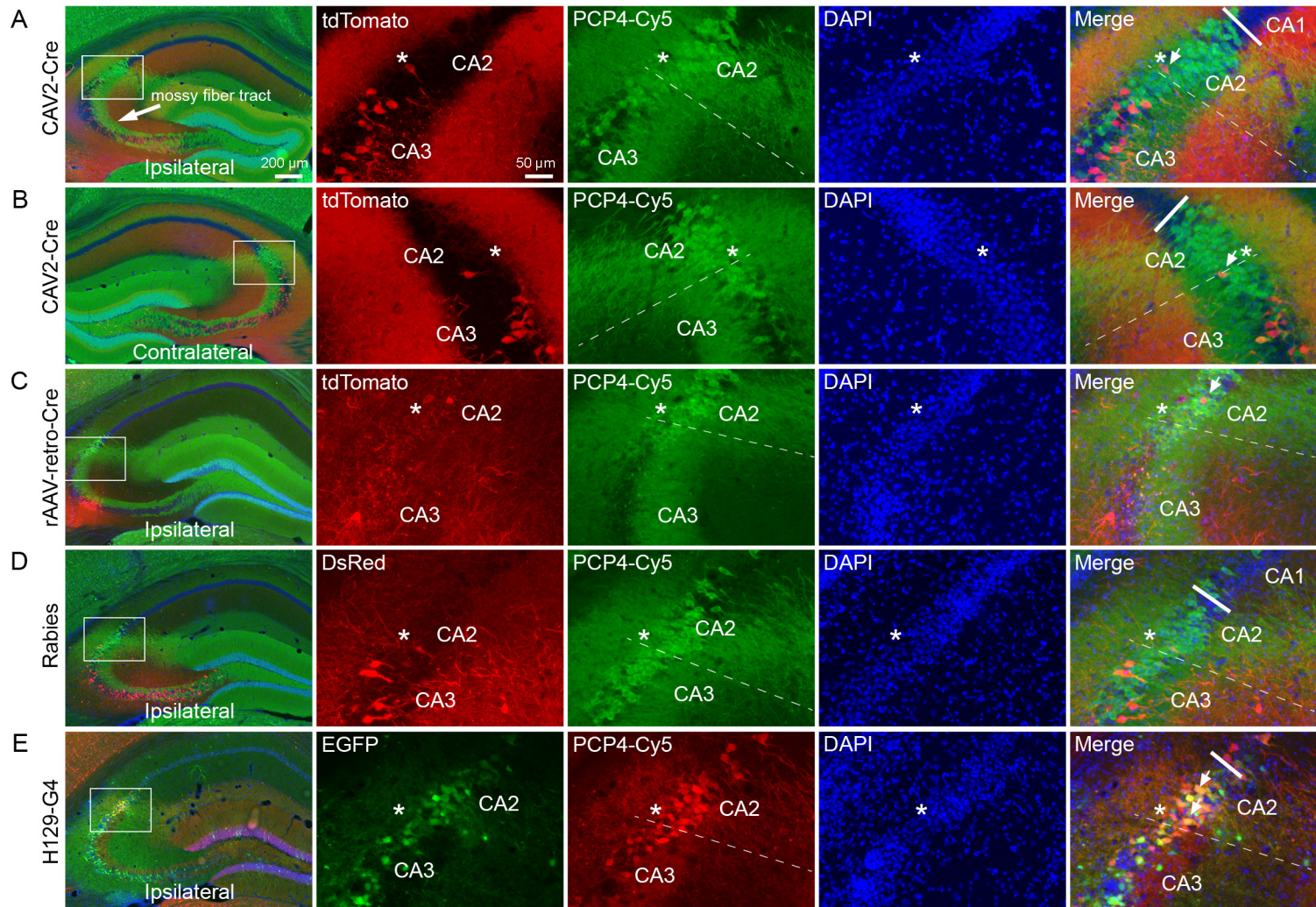

Supplement: S3 Fig — (A) From the left to right, the first panel shows a low magnification image of PCP4 staining. The second, third, and fourth panels are the enlarged views of the white boxed region in the first panel. The CAV2-Cre cells are visualized with tdTomato in the Ai9 mouse, PCP4 immunoreactivity is visualized with a Cy5-conjugated secondary antibody but presented as a green pseudo-color. DAPI staining is blue. The star and dashed line indicate the border between distal CA3 and CA2. The arrow in the fifth panel indicates the virally labeled neuron located in CA2. The solid white line indicates the CA2/CA1 border. (B–E) Example results of PCP4 immunostaining of more virally labeled brain sections, CAV2-Cre (B), rAAV-retro-Cre (C), rabies virus (D), and H129-G4 (E), following the same format as in A. Rabies-infected neurons are labeled with DsRed in D. H129-G4 infected neurons are visualized with EGFP in E. PCP4 immunoreactivity is revealed with a Cy5-conjugated secondary antibody but presented as a red pseudo-color in E. The scale bar (200 μm) applies to low magnification images in A–E; the scale bar (50 μm) applies to all the high magnification images in A–E. (PDF) [file pbio.3001127.s003.pdf]

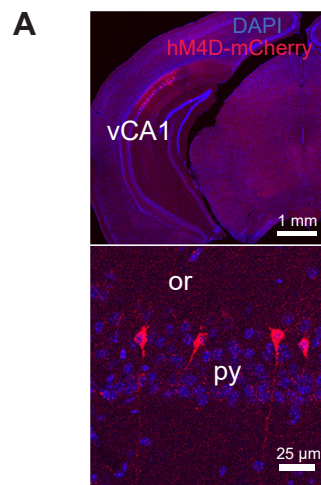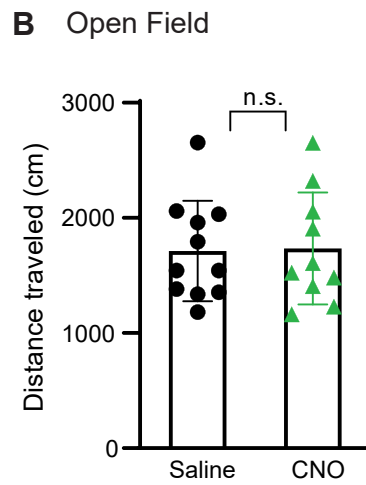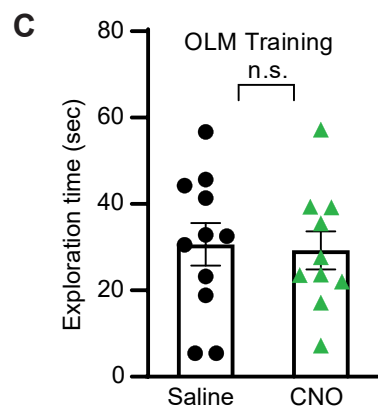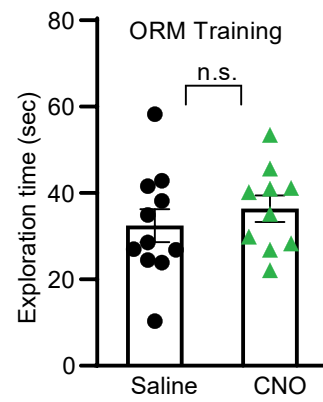

Supplement: S4 Fig — (A) Top: Example section image of hM4D-mCherry expression in vCA1, following the injection of CAV2-Cre in dCA3 and local vCA1 injection of AAV2-DIO-hM4D-mCherry. Bottom: The high magnification image of a portion of vCA1 shows that AAV-hM4D-infected neurons are localized in the pyramidal layer. DAPI staining is blue. (B) Locomotor activity in the open field box. The total distance traveled in the open arena in 10 minutes is presented (n = 11 mice for saline control, n = 10 mice for CNO treatment). The group average data are represented mean ± SE. Control and CNO treated mice did not show significant differences in their total traveling distance in the open field (no significance, n.s., p > 0.999, Mann–Whitney U test). (C) Exploration times for the 2 objects during the training session in OLM and ORM tests. Left, the total exploration time spent at object 1 and object 2 in the OLM training session (no significance, n.s., p = 0.917, Mann–Whitney U test). Right, the total exploration time spent at object 1 and object 2 in the ORM training session (no significance, n.s., p = 0.972, Mann–Whitney U test). n = 11 mice for saline control, n = 10 mice for CNO treatment. The raw data for S4B and S4C Fig are included in S3 Data. CNO, clozapine N-oxide; OLM, object location memory; or, oriens layer; ORM, object recognition memory; py, pyramidal layer; vCA1, ventral CA1. (PDF) [file pbio.3001127.s004.pdf]

## A vCA1

AP: -3.02 mm

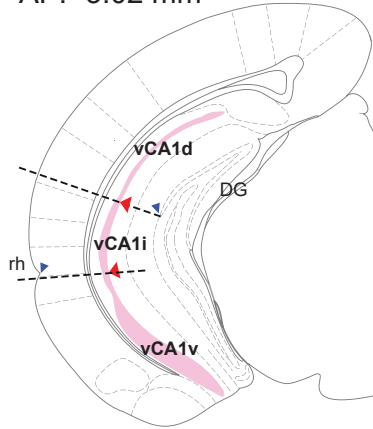

AP: -3.16 mm

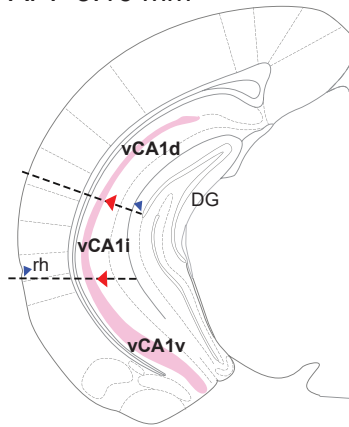

AP: -3.28 mm

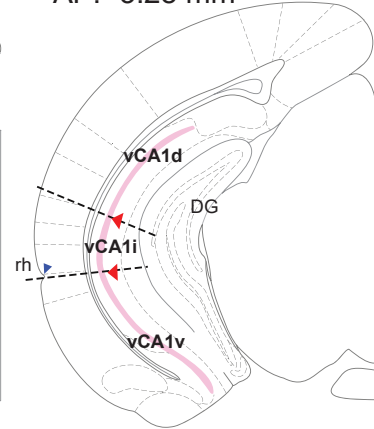

## B SUBv

AP: -3.88 mm

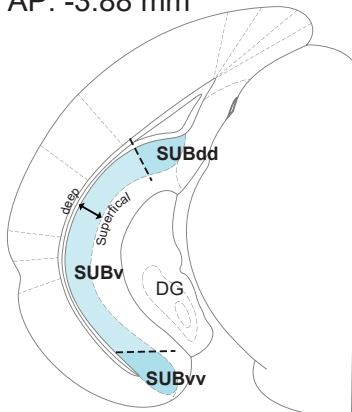

AP: -4.04 mm

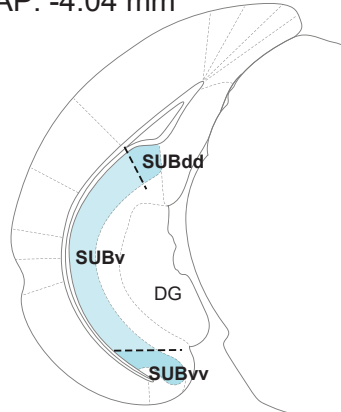

## C SUBtr

AP: -4.16 mm

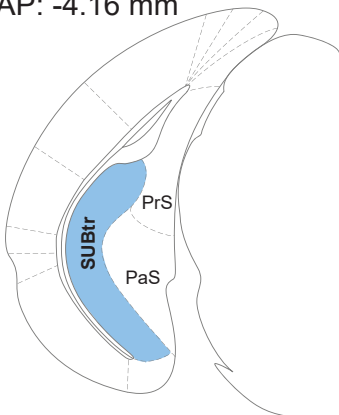

AP: -4.24 mm

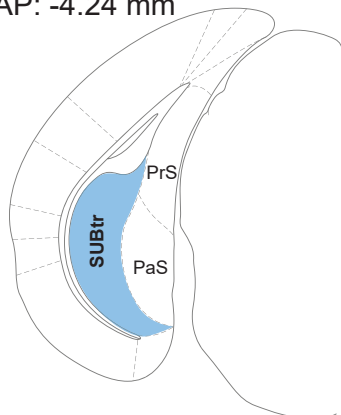

AP: -4.36 mm

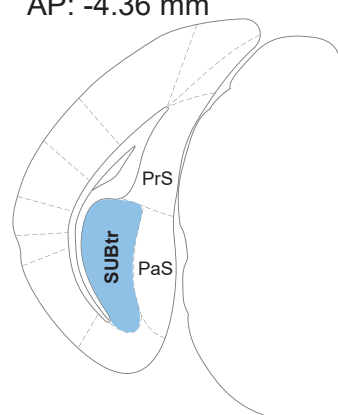

Supplement: S5 Fig — (A) Schematic illustrations of vCA1 at different AP locations. The red arrowheads divide vCA1 into 3 subdivisions, dorsal (vCA1d), intermediate (vCA1i), and ventral (vCA1v). The blue arrowhead indicates the ventral edge of the DG lateral blade and the dorsal edge of the rhinal fissure (rh). The dashed lines indicate the boundaries between these subdivisions. The pink label shows the pyramidal layer of vCA1. (B) and (C) show delineations of the subregions of SUB complex (SUBdd, SUBv, SUBvv, and SUBtr). AP, anterior–posterior; DG, dentate gyrus; Pas, parasubiculum; Prs, presubiculum; SUB, subiculum; SUBtr, subiculum transition area; SUBv, ventral subiculum. (PDF) [file pbio.3001127.s005.pdf]
