# Supplementary material for: Noncanonical projections to the hippocampal CA3 regulate spatial learning and memory by modulating the feedforward hippocampal trisynaptic pathway
Source: PLoS Biol. 2021 Dec 20;19(12):e3001127. doi: 10.1371/journal.pbio.3001127 (PMC8741299; doi:10.1371/journal.pbio.3001127)
Supplement: S1 Table — (PDF) [file pbio.3001127.s006.pdf]

Supplementary Table 1: Mouse strains and viral injections

Target Region	Mouse Strain	Coordinates (mm)	Virus	Viral Injection Method	Experiments performed
CA3a	Camk2a-Cre; TVA (N=3)	ML: -2.48; AP: -1.94; DV: -2.24	AAV8-EF1a-DIO-H2B-GFP-2A-OG<br>EnvA-SADΔG-RV-DsRed	Iontophoretic<br>Pressure	Retrograde monosynaptic rabies tracing of inputs to excitatory neurons in dorsal CA3a.
	Camk2a-Cre (N=3)		AAV8-hSyn-DIO-TC66T-2A-EGFP-2A-OG<br>EnvA-SADΔG-RV-DsRed	Iontophoretic<br>Pressure	
CA3b	Camk2a-Cre; TVA (N=3)	ML: -1.97; AP: -1.94; DV: -2.24	AAV8-EF1a-DIO-H2B-GFP-2A-OG<br>EnvA-SADΔG-RV-DsRed	Iontophoretic<br>Pressure	Retrograde monosynaptic rabies tracing of inputs to excitatory neurons in dorsal CA3b.
	Camk2a-Cre (N=3)		AAV8-hSyn-DIO-TC66T-2A-EGFP-2A-OG<br>EnvA-SADΔG-RV-DsRed	Iontophoretic<br>Pressure	
CA3c	Camk2a-Cre; TVA (N=2)	ML: -1.92; AP: -2.06; DV: -2.13	AAV8-EF1a-DIO-H2B-GFP-2A-OG<br>EnvA-SADΔG-RV-DsRed	Iontophoretic<br>Pressure	Retrograde monosynaptic rabies tracing of inputs to excitatory neurons in dorsal CA3c.
	Camk2a-Cre (N=4)		AAV8-hSyn-DIO-TC66T-2A-EGFP-2A-OG<br>EnvA-SADΔG-RV-DsRed	Iontophoretic<br>Pressure	
CA3b	Ai9 (RCL-tdTomato) (N=6)	ML: -1.97; AP: -1.94; DV: -2.24	CAV2-Cre	Pressure	Retrograde CAV2-Cre tracing of excitatory inputs to dorsal CA3.
CA3b	Ai9 (RCL-tdTomato) (N=5)	ML: -1.97; AP: -1.94; DV: -2.24	rAAV2-retro-Cre	Pressure	Retrograde rAAV-retro-Cre tracing of excitatory inputs to dorsal CA3.

vCA1	C57BL/J6 (N=4)	ML: -3.5; AP: -3.28; DV: -3.11	HSV (H129-G4) & Retrobeads (red)	Pressure	Anterograde HSV-mediated monosynaptic tracing of vCA1-CA3 projection. Retrobeads are used for verifying the injection site.
SUBv	C57BL/J6 (N=2)	ML: -3.22; AP: -4.16; DV: -3.45	HSV (H129-G4) & Retrobeads (red)	Pressure	Anterograde HSV-mediated monosynaptic tracing of SUBv-CA3 projection. Retrobeads are used for verifying the injection site.
CA3a/ vCA1	C57BL/6J (N=21)	ML: ±2.48; AP: -1.94; DV: -2.24<br>ML: ±3.5; AP: -3.28; DV: -3.11	CAV2-Cre injection in dorsal CA3a<br>AAV2-DIO-hM4D-mcherry injection in vCA1	Pressure	Genetic inactivation of dCA3-projecting vCA1 neurons with dual viral injection.

Note that for all experiments, we used mice of either sex between 4-6 months old. While more mice were used for experiments, the “N” indicated only reflects the verified cases that are up to our inclusion criteria and included for analysis .
