# Supplementary material for: Noncanonical projections to the hippocampal CA3 regulate spatial learning and memory by modulating the feedforward hippocampal trisynaptic pathway
Source: PLoS Biol. 2021 Dec 20;19(12):e3001127. doi: 10.1371/journal.pbio.3001127 (PMC8741299; doi:10.1371/journal.pbio.3001127)
Supplement: S2 Table — (PDF) [file pbio.3001127.s007.pdf]

Supplementary Table 2. Quantitative input strengths of Camk2α-Cre excitatory neurons in the CA3 subregions.

a. CA3a data

**CA3a**	MS-DBB	Contra dCA3	Contra vCA3	GrDG	Hilus	LEC	MEC	MnR	RM	Prh	vCA1 py.	vCA1 or.	vCA1 py/or	SUBv	SUBtr	SUBv/SUBtr
Average **CSI**	1.556	3.823	5.995	2.903	1.215	1.992	3.645	0.033	0.039	0.181	9.351	0.328	9.678	1.411	2.024	3.435
SE	0.646	2.250	2.342	1.023	0.668	1.213	2.224	0.027	0.033	0.164	5.111	0.125	5.200	0.857	1.483	2.336
Average **PI**	0.058	0.162	0.205	0.130	0.030	0.046	0.075	0.003	0.003	0.005	0.227	0.014	0.241	0.031	0.037	0.068
SE	0.020	0.071	0.063	0.048	0.010	0.013	0.028	0.002	0.002	0.003	0.066	0.005	0.065	0.010	0.014	0.024

**Average # of starter cells per section for each case**	3, 3, 3, 3, 10, 5	**Total input mapped neurons outside ipsilateral CA3**	319 ± 79.8 per case	**N = 6 cases**

b. CA3b data

**CA3b**	MS-DBB	Contra dCA3	Contra vCA3	GrDG	Hilus	LEC	MEC	MnR	RM	Prh	vCA1 py.	vCA1 or.	vCA1 py/or	SUBv	SUBtr	SUBv/SUBtr
Average **CSI**	2.307	11.029	11.249	6.361	0.449	0.575	1.001	0.025	0.034	0.231	0.259	0.115	0.375	0.025	0.026	0.051
SE	0.648	5.233	7.458	1.646	0.128	0.238	0.292	0.015	0.020	0.090	0.114	0.047	0.108	0.013	0.014	0.025
Average **PI**	0.092	0.286	0.203	0.244	0.032	0.026	0.050	0.001	0.001	0.012	0.038	0.005	0.043	0.004	0.004	0.008
SE	0.017	0.024	0.071	0.066	0.011	0.007	0.021	0.0005	0.001	0.005	0.029	0.002	0.030	0.003	0.003	0.006

**Average # of starter cells per section for each case**	5, 21, 9, 13, 8, 4	**Total input mapped neurons outside ipsilateral CA3**	953 ± 234.7 per case	**N = 6 cases**

c. CA3c data

**CA3c**	MS-DBB	Contra dCA3	Contra vCA3	GrDG	Hilus	LEC	MEC	MnR	RM	Prh	vCA1 py.	vCA1 or.	vCA1 py/or	SUBv	SUBtr	SUBv/ SUBtr
Average **CSI**	2.603	4.910	1.193	7.248	1.355	0.526	3.743	0.072	0.048	0.942	0.073	0.048	0.121	0.002	0.011	0.013
SE	0.703	1.560	0.624	2.577	0.543	0.145	1.431	0.059	0.022	0.394	0.047	0.041	0.054	0.002	0.008	0.008
Average **PI**	0.108	0.242	0.057	0.302	0.052	0.024	0.162	0.003	0.002	0.038	0.005	0.002	0.008	0.0003	0.001	0.001
SE	0.011	0.056	0.022	0.066	0.016	0.006	0.061	0.002	0.001	0.011	0.004	0.002	0.005	0.0003	0.0007	0.0009

**Average # of starter cells per section for each case**	17, 12, 3, 4, 8, 2	**Total input mapped neurons outside ipsilateral CA3**	374 ± 88.7 per case	**N = 6 cases**

Note that the input connection strength index (CSI) is defined as the ratio of the number of presynaptic neurons in a given brain structure versus the number of starter neurons measured (see Methods). The proportion of inputs (PI) index is defined as the number of labeled neurons in a given brain structure compared to the total number of labeled neurons from all input mapped regions (see Methods). The local ipsilateral CA3 input quantification is excluded. All data is presented as mean ± SE. Abbreviations: Contra dCA3, contralateral dorsal CA3; Contra vCA3, contralateral ventral CA3; GrDG, granule cell layer of dentate gyrus; Hilus, dentate hilus; LEC, lateral entorhinal cortex; MEC, medial entorhinal cortex; MnR, median raphe nucleus; MS-DBB, medial septum and diagonal band of Broca (including horizontal and vertical diagonal band); Prh, perirhinal cortex; RM, retromammillary nucleus; SUBtr, subiculum transition area; SUBv, designated ventral subiculum; vCA1 py., pyramidal cell layer of ventral CA1; vCA1 or., oriens cell layer of ventral CA1.
