# Supplementary material for: Noncanonical projections to the hippocampal CA3 regulate spatial learning and memory by modulating the feedforward hippocampal trisynaptic pathway
Source: PLoS Biol. 2021 Dec 20;19(12):e3001127. doi: 10.1371/journal.pbio.3001127 (PMC8741299; doi:10.1371/journal.pbio.3001127)
Supplement: S3 Table — (PDF) [file pbio.3001127.s008.pdf]

Supplementary Table 3. Statistical comparisons of input strengths to excitatory neurons in CA3 subregions.

Comparison of the Connection of Strength Index (CSI)

Brain region	Kruskal-Wallis test		Dunn's multiple comparison		
			CA3a vs. CA3b	*CA3a vs. CA3c*	*CA3b vs. CA3c*
	H	p value	Adjusted-p value	Adjusted-p value	Adjusted-p value
MS-DBB	1.556	0.484	ns	ns	ns
Contra dCA3	2.211	0.353	ns	ns	ns
Contra vCA3	3.404	0.190	ns	ns	ns
GrDG	3.170	0.217	ns	ns	ns
Hilus	1.058	0.614	ns	ns	ns
LEC	0.512	0.787	ns	ns	ns
MEC	1.064	0.623	ns	ns	ns
MnR	0.201	0.926	ns	ns	ns
RM	0.353	0.858	ns	ns	ns
Prh	5.780	**0.049**	ns	ns	ns
vCA1 py.	6.301	**0.036**	ns	**0.037**	ns
vCA1 or.	5.923	**0.046**	ns	**0.005**	ns
vCA1 py./or.	10.270	**0.002**	ns	**0.004**	ns
SUBv	8.273	**0.009**	ns	**0.014**	ns
SUBtr	9.198	**0.005**	ns	**0.014**	ns
SUBv/SUBtr	9.173	**0.006**	ns	**0.009**	ns

Comparison of Proportion of Inputs (PI)

Brain region	Kruskal-Wallis test		Dunn's multiple comparison		
			CA3a vs. CA3b	*CA3a vs. CA3c*	*CA3b vs. CA3c*
	H	p value	Adjusted-p value	Adjusted-p value	Adjusted-p value
MS-DBB	4.819	0.086	ns	ns	ns
Contra dCA3	4.819	0.086	ns	ns	ns
Contra vCA3	4.713	0.092	ns	ns	ns
GrDG	3.930	0.142	ns	ns	ns
Hilus	1.109	0.595	ns	ns	ns
LEC	1.556	0.484	ns	ns	ns
MEC	2.117	0.367	ns	ns	ns
MnR	0.130	0.956	ns	ns	ns
RM	0.032	0.990	ns	ns	ns
Prh	6.604	**0.035**	ns	**0.031**	ns
vCA1 py.	7.193	**0.020**	ns	**0.023**	ns
vCA1 or.	4.777	0.088	ns	ns	ns
vCA1 py./or.	8.879	**0.006**	ns	**0.010**	ns

SUBv	7.462	**0.019**	ns	**0.020**	ns
SUBtr	10.960	**0.001**	ns	**0.007**	ns
SUBv/SUBtr	10.100	**0.002**	ns	**0.006**	ns

Note that bold values indicate statistically significant differences with $p < 0.05$. “ns” indicates no significant difference in the comparison test. Brain regional abbreviations are the same as in Table 2. N=6 cases each per CA3 subregion, CA3a, CA3b and CA3c.
