# Supplementary material for: Noncanonical projections to the hippocampal CA3 regulate spatial learning and memory by modulating the feedforward hippocampal trisynaptic pathway
Source: PLoS Biol. 2021 Dec 20;19(12):e3001127. doi: 10.1371/journal.pbio.3001127 (PMC8741299; doi:10.1371/journal.pbio.3001127)
Supplement: S4 Table — (PDF) [file pbio.3001127.s009.pdf]

Supplementary Table 4: The numbers of labeled neurons in CA3 input mapped regions

Virus	Injection region	MS-DBB	Contra dCA3	Contra vCA3	GrDG	Hilus	LEC	MEC	MnR	RM	Prh	vCA1d	vCA1i	SUBv	SUBtr	**Total counts**
CAV2-Cre	CA3	825	1385	502	50	479	1920	1365	38	169	6	1002	393	1305	71	9510
CAV2-Cre	CA3	396	1000	306	40	406	1607	1068	15	131	4	568	58	733	755	7087
CAV2-Cre	CA3	228	1317	365	6	59	930	76	26	75	4	256	102	173	108	3725
CAV2-Cre	CA3	427	1588	509	7	305	571	723	20	65	3	499	224	403	354	5698
CAV2-Cre	CA3	113	155	27	1	2	269	551	4	21	0	115	47	151	60	1516
CAV2-Cre	CA3	134	155	42	5	4	410	400	2	58	1	140	59	136	92	1638
rAAV-retro-Cre	CA3	422	1550	489	5960	1436	2479	5182	220	268	40	4107	601	3736	1079	27569
rAAV-retro-Cre	CA3	301	597	220	2288	18	982	1571	74	172	35	3992	277	1620	401	12548
rAAV-retro-Cre	CA3	150	407	43	1664	26	1102	1786	144	91	26	2355	50	429	305	8578
rAAV-retro-Cre	CA3	418	1207	514	6160	1159	2775	3871	180	238	18	1903	483	1248	656	20830
rAAV-retro-Cre	CA3	28	74	10	576	207	75	77	44	29	0	36	5	12	6	1179

Virus	Injection region	MS-DBB	Contra dCA3	Contra vCA3	GrDG	Hilus	LEC	MEC	MnR	RM	Prh	vCA1 py.	vCA1 or.	SUBv	SUBtr	**Total counts**
Rabies	CA3a	2	6	3	29	0	5	11	0	1	0	31	3	4	5	100
Rabies	CA3a	5	19	25	43	6	17	32	0	0	0	61	0	7	6	221
Rabies	CA3a	23	8	43	12	22	39	71	0	0	5	169	4	28	47	471
Rabies	CA3a	6	5	37	1	8	2	5	1	0	0	43	1	6	4	119
Rabies	CA3a	62	143	99	82	9	17	0	3	3	8	20	12	0	2	460
Rabies	CA3a	29	225	239	30	9	3	1	0	0	0	0	4	1	3	544

Rabies	CA3b	35	302	418	84	0	2	9	0	1	1	1	3	0	0	856
Rabies	CA3b	137	345	101	689	60	34	92	4	2	40	25	2	6	0	1537
Rabies	CA3b	35	72	66	18	14	8	9	0	0	2	52	4	5	6	291
Rabies	CA3b	77	264	17	247	69	54	138	2	6	27	29	5	2	3	940
Rabies	CA3b	63	98	46	166	9	7	7	0	0	0	0	1	0	0	397
Rabies	CA3b	108	613	517	299	17	54	60	3	0	18	3	5	0	1	1698
Rabies	CA3c	44	192	51	157	21	18	75	2	0	24	16	3	1	2	606
Rabies	CA3c	57	299	101	174	6	9	17	1	2	3	0	6	0	0	675
Rabies	CA3c	17	26	12	10	17	9	77	0	1	12	0	0	0	0	181
Rabies	CA3c	39	53	5	84	26	8	70	4	1	15	0	0	0	0	305
Rabies	CA3c	41	98	7	158	3	2	10	0	0	1	0	0	0	1	321
Rabies	CA3c	22	11	1	75	13	3	22	0	0	10	1	0	0	0	158

Note that cell counts are based on the quantification of virally labeled cells in our brain section series (every one out of three sections, in 30 μm section thickness). The total cell counts are positively correlated with the numbers of starter neurons in CA3 subregions. See Supplementary Table 2 for details.
